# Configuration determination by residual dipolar couplings: accessing the full conformational space by molecular dynamics with tensorial constraints†
†Electronic supplementary information (ESI) available. See DOI: 10.1039/c9sc01084j


**DOI:** 10.1039/c9sc01084j

**Published:** 2019-07-29

**Authors:** Pavleta Tzvetkova, Ulrich Sternberg, Thomas Gloge, Armando Navarro-Vázquez, Burkhard Luy

**Affiliations:** a Institute of Organic Chemistry and Institute for Biological Interfaces 4 – Magnetic Resonance , Karlsruhe Institute of Technology (KIT) , Fritz-Haber-Weg 6 , 76131 Karlsruhe , Germany . Email: armando.deus@gmail.com ; Email: burkhard.luy@kit.edu

## Abstract

Residual dipolar couplings (RDCs) and other residual anisotropic NMR parameters provide valuable structural information of high quality and quantity, bringing detailed structural models of flexible molecules in solution in reach. The corresponding data interpretation so far is directly or indirectly based on the concept of a molecular alignment tensor, which, however, is ill-defined for flexible molecules. The concept is typically applied to a single or a small set of lowest energy structures, ignoring the effect of vibrational averaging. Here, we introduce an entirely different approach based on time averaged molecular dynamics with dipolar couplings as tensorial orientational restraints that can be used to solve structural problems in molecules of any size without the need of introducing an explicit molecular alignment tensor into the computation. RDC restraints are represented by their full 3D interaction tensor in the laboratory frame, for which pseudo forces are calculated using a secular dipolar Hamiltonian as the target. The resulting rotational averaging of each individual tensorial restraint leads to structural ensembles that best fulfil the experimental data. Using one-bond RDCs, the approach has been implemented in the force field procedures of the program COSMOS and extensively tested. A concise theoretical introduction, including the special treatment of force fields for stable and fast MD simulations, as well as applications regarding configurational analyses of small to medium-sized organic molecules with different degrees of flexibility, is given. The observed results are discussed in detail.

## Abstract 2

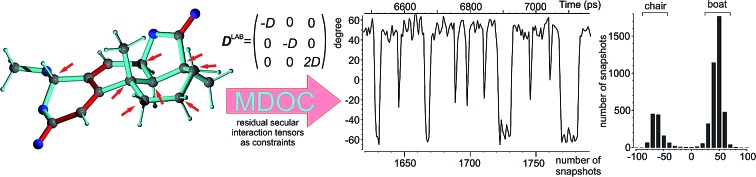
The use of tensorial orientational constraints for NMR-derived residual dipolar couplings (RDCs) in molecular dynamics simulations brings detailed structural models of flexible molecules in solution in reach.

## 1 Introduction

Residual dipolar couplings (RDCs) are an efficient tool for the determination of the relative configuration of small organic molecules.^1^–^5^ The use of RDCs has been boosted by the availability of weak aligning media compatible with standard organic solvents such as CDCl_3_ 6–10 or DMSO-d_6_,^11^–^18^ and most other common NMR solvents.^19^–^22^ While it is widely recognized that residual anisotropic NMR parameters are suited for the determination of dynamics in biomolecules,^23^,^24^ they are equally amenable to the structure elucidation of small molecules with inherent flexibility. However, physically sound data interpretation is difficult, especially in the case of small to medium sized molecules in which typically only a single alignment medium is employed and, although in principle a multitude of internuclear couplings exists, the amount of practically accessible RDCs is limited. Vibrations and slow conformational changes in molecules significantly complicate the situation. For small, rigid molecules simple harmonic modes can be obtained from DFT calculations and have been successfully applied,^25^ but for most cases the corresponding contributions are either neglected or treated using considerable approximations. Here, instead, we introduce molecular dynamics with RDC-based orientational tensorial constraints applied in the laboratory frame as a physically sound method for the determination of relative configurations of molecules with inherent flexibility.

Since RDCs, in contrast to locally determined NMR parameters like NOEs or *J*-couplings, depend on the global orientation of the internuclear vectors with respect to the external magnetic field, they provide information that can become crucial for the solution of relative configuration problems, in particular in the case of stereogenic centres too distant from each other. The technique has been successfully applied to the structure elucidation of several natural^26^–^33^ and synthetic compounds.^34^–^37^ In the case of rigid molecules, the analysis is based on fitting the experimental data to global molecular order or alignment tensors **S** for each possible configuration, which are then ranked according to the fitting between the experimental and back computed values. This fitting is accomplished in most of the cases by a least squares solution of a set of linear equations using singular value decomposition (SVD)^38^ – although other techniques can be employed.^38^–^45^ Nevertheless, in many cases the studied compounds have some degree of conformational flexibility which needs to be taken into account when analysing the data. It has been known for decades that vibrational corrections need to be applied for accurate RDC interpretation,^46^,^47^ as the full enthalpically and entropically driven conformational space contributes to the alignment. But these contributions are difficult to include in everyday applications. The currently employed approaches are mostly based on fitting RDC data to a discrete ensemble of lowest energy conformations obtained typically by a force-field-based stochastic conformational search, a procedure that we will call here the “static” approximation. Other physically more sound models involve the detailed treatment of a defined, limited set of flexible bonds, or the use of restrained molecular dynamics (r-MD) methodologies, usually applied with RDCs as scalar restraints in an alignment tensor frame of reference.

The “static” method uses in most cases the “single tensor” approximation,^48^–^50^ *i.e.* the assumption that different conformers share a common global molecular order tensor, which is mostly called the Saupe tensor or alignment tensor (we refer to this method throughout the paper as the alignment tensor method). Sharing a common order tensor, the populations of different conformations can be estimated through different optimization techniques.^43^,^50^–^52^ Although the procedure is based on a rough approximation, its use is reasonable when the conformational changes do not cause a significant change of the global shape of the molecule. As only low energy conformers are included in the data interpretation, experimental RDCs are practically never fulfilled within the experimental error. The use of the single tensor approximation requires the definition of a common frame for all conformations, which is usually accomplished by overlay of the atomic coordinates,^48^,^50^,^51^ but which is generally ill-defined in the presence of flexibility.

Static multi tensor approaches, where an order tensor is determined for each conformation considered, have been scarcely used in structure determination problems since the large number of unknown parameters to be fitted makes the problem usually bad conditioned. Vibrational averaging is not considered and the populations of different conformations cannot be determined, unless a common degree of order is assumed for all determined tensors.^50^,^53^ Alternatively, populations can be derived from other NMR constraints such as scalar couplings or NOE-derived distances.^29^ Another approach is based on the maximum entropy (ME) method that attempts to explain the experimental data with the bare minimum of information, therefore maximizing the freedom on the rotational global and internal degrees of freedom. The original unconstrained ME^54^ frequently leads to too flat conformational spaces, and in fact wrongly predicts a complete freedom around internal coordinates in the isotropic limit. This is due to the fact that the method ignores the enthalpy contributions to the total entropy coming from the different potential energies of different conformational states. The method has also been extended to include all kinds of constraints to obtain a minimum set of conformations to fulfil sparse experimental data and to include *a priori* structural information.^55^

Based on the Marcelja molecular mean-field approximation,^56^ a successful and physically sound approach to the flexibility problem is the additive potential (AP) method,^57^ which is very well suited for the analysis of torsional distributions in simple molecules. The method requires the definition of a functional form for the isotropic limit conformational distribution and allows the separate treatment of molecular fragments. It has been considerably enhanced by the introduction of Gaussian angular distributions into the so-called AP-DPD approach.^46^,^57^ A clear comparison of the AP and ME methods was reported by Emsley *et al.*^58^ However, the method unfortunately is computationally demanding, currently limiting the application to systems with less than 60 atoms. The combined APME method proposed by Maliniak and coworkers combines the two methods and avoids the use of a functional form for the isotropic limit distribution by simultaneous fitting to *J*-coupling and NOE distances.^59^,^60^

All methods described so far are based on providing a single structure or an ensemble of structures with the lowest energies that is used for fitting experimental data to a single tensor or a sum of individual alignment tensors. More sophisticated approaches incorporate vibrational averaging based on harmonic approximations to further enhance the fitting procedure. With the exception of a number of quite small molecules, however, the amount of accessible experimental parameters does not match the number of fitting parameters, which invokes additional approximations like the ME approach. Very different from this procedure, the conformationally accessible space, including excited states and entropic contributions, is inherently sampled in restrained molecular dynamics (r-MD) approaches, where RDCs are used as constraints.

In most cases, RDCs as scalar constraints are introduced in steepest descent or r-MD simulations based on protocols using axial and rhombic parameters of an alignment tensor that has been estimated initially and RDCs were used as angular as well as combined angular and distance restraints.^61^–^65^ A clear advantage is the direct combination with other NMR observables such as scalar couplings or NOE-derived distances as constraints.^26^,^37^,^66^ r-MD simulations have been used in combination with floating chirality protocols^67^,^68^ which allow the computation to directly lead to the best fitting configuration.^69^–^71^ A fundamental problem with all scalar procedures, however, is the approximation to a single alignment tensor in a molecular frame of reference, which *per se* is potentially a rough estimate. The obtained ensembles might fulfil experimental constraints, but at the same time not resemble the actually physically existing structural ensembles.

Molecular dynamics and Monte Carlo-type calculations have also been used in different ways to characterize intrinsically disordered proteins as a highly flexible class of molecules. The flexible meccano approach^72^ uses an unrestrained approach for generating a structural ensemble for which populations are refined using the prediction of an alignment tensor for each conformation, similar to the tramite approach;^42^ and finally the ϑ-method^73^–^75^ uses angles derived from RDCs as relative scalar constraints for the refinement of structures in the laboratory frame, where a principle of replica-averaging in the framework of the maximum entropy is used in line with a linear scaling factor to replace the alignment tensor.

In a radically different MD approach Sternberg *et al.*^76^–^78^ have proposed the analysis of anisotropic NMR properties, such as quadrupolar couplings, chemical shifts or dipolar couplings, by performing molecular dynamics with orientational constraints (MDOC). This method is based on tensorial constraints which individually have to fulfil the secular dipolar interaction Hamiltonian in the laboratory frame without the assumption of an overall order or alignment tensor. It has been originally applied to the analysis of ^2^H quadrupolar splitting in several membrane-bound peptides.^79^,^80^ In the present work we demonstrate on a number of molecules with different complexities how this procedure can be applied to the analysis of RDC data and the determination of relative configuration in small molecules. As the approach does not make any assumptions on the molecular shape or the corresponding overall alignment, it can equally be applied to any kind of molecule and lead to structural ensembles that fully comply with the experimental data as vibrational averaging and other conformational variabilities are included in the calculation.

## 2 Theory

In the following we will briefly review the MDOC methodology with an emphasis on the analysis of one bond ^1^*D*_CH_ RDCs and special extensions to data originating from weakly aligned molecules. Due to its dependence on the relative orientation between the internuclear vector and the external magnetic field, the dipolar coupling between two nuclei *i* and *j* in the laboratory frame is expressed as a second rank symmetric tensor **D**^L^(*i*,*j*). In the laboratory frame, the *z*-axis is assumed to be parallel to the **B**_0_ external field, and at an arbitrarily chosen time point *t*_0_ the components of **D**^L^(*i*,*j*) in rad/s are of the form
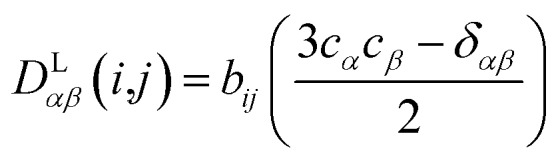

1
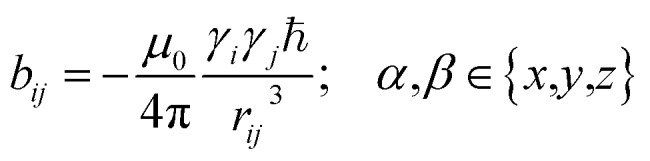
where *r*_*ij*_ is the distance between the nuclei *i* and *j*, *γ*_*i*_ and *γ*_*j*_ are the corresponding gyromagnetic ratios, *c*_*α*_ and *c*_*β*_ are the Cartesian components of the **r**_*ij*_ internuclear unit vector, and *δ*_*αβ*_ refers to the Kronecker delta. In the high field secular approximation, the observed dipolar couplings *D* are equal to the *zz*-component of the dipolar coupling tensor *D*L*zz*(*i*,*j*).

### 2.1 Averaged dipolar couplings

Experimental dipolar couplings are always an average of the different vibrational states, conformations, and orientations being populated during the course of the measurement. Restricting ourselves to dipolar couplings between directly bonded nuclei, we will neglect the stretching components of vibrations, as the corresponding changes in the bond length are very small and occur on the order of femtoseconds and can be taken care of by a stretching averaged distance *r*_*ij*_. All other contributions lead to changes in orientation that will be treated in the following manner.

The preferred dipolar coupling associated coordinate system is chosen in such a way that the new *z*′-axis points along the vector **r**_*ij*_ and the dipolar coupling tensor has a diagonal representation according to2
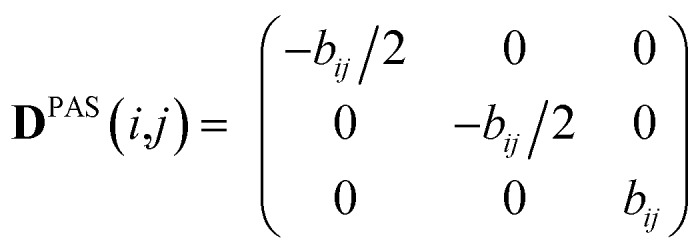



The tensorial components in this principal axis system (PAS) are transformed into the laboratory frame by a rotation matrix **T**(*i*,*j*). This transformation is expressed by a double sum over the components of the transformation matrix *T*_*αβ*_ (coupling sites *i* and *j* are omitted)3
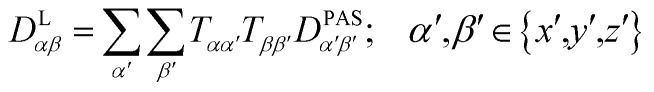



Due to rotational diffusion and other contributions, the orientation of the internuclear vector with respect to the external field changes with time and, therefore, the rotation matrix **T** becomes time dependent. In the case of partially aligned samples, where such rotations are dominated by rotational tumbling – that takes place on the order of the correlation time *τ*_c_, *i.e.* picoseconds to nanoseconds – dipolar couplings are observed as significantly downscaled time averaged values called residual dipolar couplings (RDCs). Considering a rigid molecule, the averaging of the *zz*-component of the dipolar coupling tensor can be described by an overall order matrix, the so-called Saupe matrix or alignment tensor,^81^,^82^ according to4
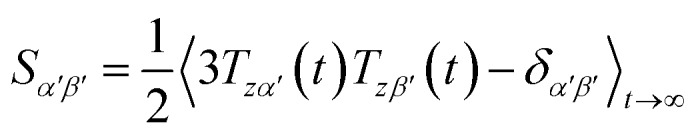

5
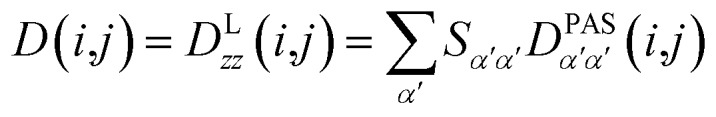



However, RDCs may be further averaged by conformational dynamics. Therefore, the products of the rotation matrix elements *T*_*αα′*_*T*_*ββ′*_ contain valuable information not only about the global molecular rotational tumbling but also about the typically much slower internal motions. In this context it is of practical use to introduce local (segmental) order tensors **S**(*i*,*j*) for each coupling in the molecule with the components6
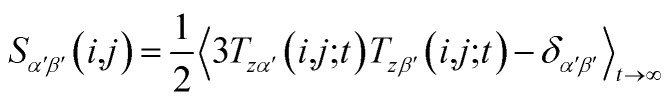
where the dipolar couplings *D* are represented by7
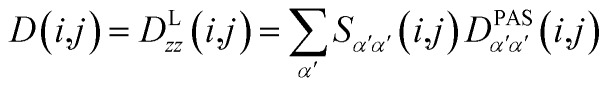
which contain vibrational, conformational, and overall rotational tumbling time averaging up to time *t* (denoted within brackets) for bond vectors **r**_*ij*_. Note that since in the secular approximation only the diagonal component of the dipolar coupling tensor contributes to the observed couplings and the *zz*-component is fully sufficient for description, the summation runs only over the *z*-components of the rotation matrices. In an MD simulation, the segmental order tensors are approximated by all orientations in the corresponding trajectory, leading to finite averaging over time *t*. The local order tensors sufficiently describe the averaged *zz*-component of the dipolar coupling tensor corresponding to the measured RDCs, but they do not make full use of the tensorial properties of RDCs as restraints. With rotation matrices **T** in hand, also the full dipolar coupling tensor for each individual coupling (*i*,*j*) can be averaged according to8
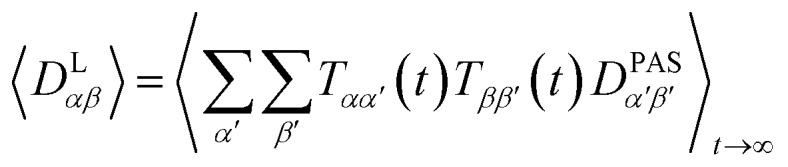



The MDOC method uses the whole coupling tensors The MDOC method uses the whole coupling tensors 〈*D*L*αβ*(*i*,*j*))〉_*t*_ averaged over the time span *t* of the MD trajectory as an approximation and utilizes them as restraints for calculating further transformation matrices at every MD step as described in the following sections.

### 2.2 Dipolar coupling tensor scaling

The approach derived in the previous sections could be used directly to simulate the time averaging of full residual dipolar coupling tensors of every spin pair *i* and *j*. However, experimental RDC data are obtained using the so-called alignment media that scale down the dipolar coupling tensors by a factor of approximately 1000 in order to maintain chemical shift dispersion in the corresponding spectra. Apparently, for the majority of the time, the molecule of interest is isotropically averaged and only a small fraction contributes to the measured RDCs. This scaling of anisotropy may conveniently be described by the factor *s*expAM. As the isotropic averaging can be neglected in the corresponding MDOC simulations, we can now introduce a scaling factor

9 1 ≥ *s*_AM_ ≥ *s*expAM

that significantly reduces the calculation time for rotational averaging, as only the reduced average dipolar coupling tensors have to be compared to the experimentally derived dipolar coupling tensors **D**^exp^(*i*,*j*) as derived below. The scaling to some extent separates the isotropic rotational diffusion contribution of the overall molecule, which does not contribute to conformational variations, from other structurally relevant rotations that are caused, for example, by conformational exchange. With the overall scaling factor *s*_AM_, the conformational motion, which only depends on the relative sizes of the corresponding RDCs, is given with a considerably increased weight in the MDOC simulation and setting the *s*_AM_ parameter to an appropriate value allows the system to achieve experimentally derived tensorial constraints in much shorter periods of time. The time averaging of dipolar coupling tensors can then be rewritten as10
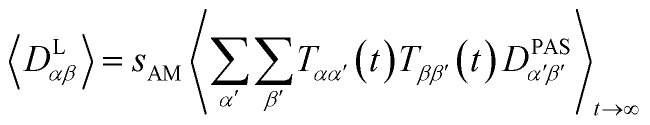



Note, however, that *s*_AM_ should not be so low that experimental couplings are not attainable in the simulations. *s*_AM_ should always be chosen a factor 2–10 higher than the largest component of an estimated Saupe matrix *S*_*αβ*_ to allow for both overall alignment of the assumedly rigid molecule and averaging of the alignment due to internal conformational changes.

### 2.3 Weighted time averaging

In simulations with constraints depending on the molecular orientations, the values of **D**^L^(*i*,*j*;*t*) are not relevant at every time step *t* and only their mean values obtained from molecular reorientations can be compared to experimental constraints. This is a general problem for all conformationally averaged NMR properties, such as NOE distance restraints or scalar couplings. Torda and van Gunsteren^114^,^115^ introduced the idea of averaging properties using an exponential decay function 
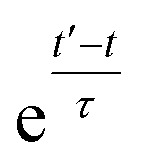
, allowing the system to “forget” past events and therefore be able to experience deviations from the instantaneous computed value to the average value. This technique, at first introduced for the averaging of NOE based distances, has been later extended to scalar coupling restraints^83^ and alignment-tensor-based scalar RDC constraints.^61^ Following this methodology, time averaged dipolar coupling tensors are given by11
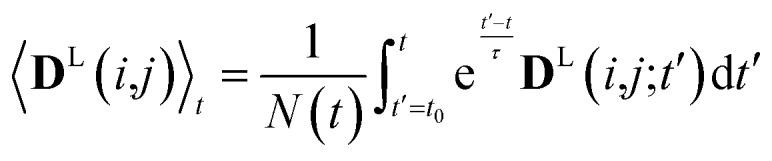



The introduction of the time average with exponential memory according to eqn (11) effectively introduces a new time scale for rotational reorientations and fluctuations. This time scale represents the lifetime of the orientation of the molecule or a mobile segment. The so-called memory time constant is denoted with *τ* and *N* is the norm of the integral. The memory time function ensures that contributions from past events are “forgotten” within the time averaging of **D**.

To further illustrate this point, an abstract system with two states A and B (representing for instance two conformers of the molecule) is considered, where in the NMR experiment the mean value of these two states is measured and used as a constraint. If the simple (arithmetic) time mean value is calculated and the system is half of the time in state A and the other half in state B, then the pseudo energy vanishes and an undefined time is needed before a difference between the constraint and calculated mean value builds up. The natural behaviour targeted in MD simulations is of a system jumping between different conformational states many times during the trajectory. In most cases, the conformational exchange has to surmount relatively large barriers and the transition events cannot be recorded in the course of standard MD procedures. Using eqn (11) we have merely to wait a time period on the order of the memory time *τ* until a difference between the constraint and time mean value develops and the pseudo forces can flip the system from A to B or *vice versa*. Therefore *τ* is the effective time scale of the accelerated MDOC simulation and we have to wait about 5*τ* before obtaining an ensemble average. Only in the case of very high barriers longer simulation times may be necessary. In practice, this can be tested by inspecting the time development of averaged dipolar couplings over a trajectory. If the average converges to a constant value, one can be certain that the overall simulation time is sufficient.

Since in conventional MD simulations the equations of motion are integrated in finite time steps Δ*t*, the integral in eqn (11) is practically implemented as a discrete sum. During the MD simulation, the sum is practically implemented as a discrete sum. During the MD simulation, the sum 〈**D**^L^(*i*,*j*))〉_(*m*+1)Δ*t*_ at time *t* = (*m* + 1)Δ*t* is calculated from the previous time step is calculated from the previous time step 〈**D**^L^(*i*,*j*))〉_*m*Δ*t*_ in a recursive manner for each time step Δ*t* according to12
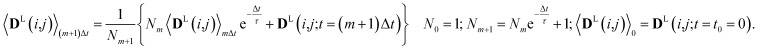



### 2.4 Pseudo energy

To carry out MD simulations driven by experimental constraints, pseudo energy terms are added to the molecular energy provided by the force field. These pseudo energies are defined as a function of the difference between the experimental and calculated tensor properties:13
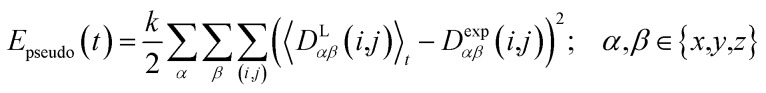
where *k* is an empirical force constant which is chosen to adjust the size and units of the pseudo energy and the sum runs over all tensor components and all spin pairs (*i*,*j*). The experimental constraints in this case are written in the tensorial form in the laboratory frame according to14
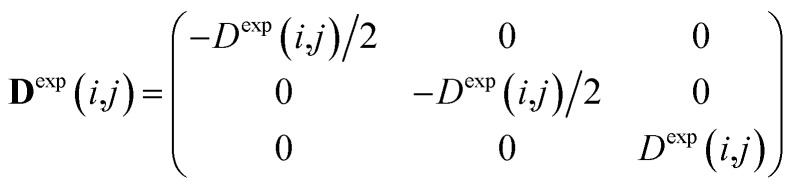
which can be derived from the measured residual dipolar couplings *D*^exp^(*i*,*j*) using the symmetry of the typical experimental setup, where both the direction of the alignment and the static magnetic field are oriented along the *z*-axis. The secular Hamiltonian is averaged from both spin and spatial conditions. In both cases the trace of the dipolar interaction must vanish, leaving diagonal *xx* and *yy*-elements to be minus half the size of the *zz*-component. Off-diagonal elements of the spin part of the Hamiltonian are zero due to the uncorrelated spin phase hypothesis of spin ensembles. In addition, the spatial part of the Hamiltonian is averaged according to the cylindrical symmetry of the alignment, which leads to an effective *D*_∞h_ or *C*_∞v_ point group. As a consequence, first the *zz*-component must represent an eigenvalue of the interaction matrix and, second, the indistinguishable *xx* and *yy* components also require zero *xy* off-diagonal elements. Especially the off-diagonal elements are important as constraints later on, as they will drive rotations of individual bonds and the whole molecule for rotational averaging. Under these secular conditions, which are certainly fulfilled over the course of an NMR experiment, real tensorial constraints for all Cartesian coordinates can be applied in the laboratory frame. In analogy to scalar constraints, every component of the tensor is used as an individual constraint and consequently the pseudo energy in eqn (13) is a sum over all tensor components, including in particular the off-diagonal elements.

### 2.5 Pseudo forces

In MD simulations, the equations of motion are solved in a discrete step by step manner and pseudo forces **F** have to be calculated from the respective pseudo energy contributions. They are obtained as derivatives of the energies with respect to the Cartesian coordinates of the atoms. In the case of orientational pseudo forces, we have to derive the transformation matrices **T**(*i*,*j*) with respect to the coordinates of the atoms that were used in their definition (for details see *e.g.* Sternberg *et al.*^77^). The transformation matrices are constructed from the unit vectors that define the actual orientation of the dipolar systems: the unit vector **e**_*z*′_ = **e**(*i*,*j*) points along the direction of the nuclei *i* and *j* (*i.e.* along the C–H bond direction) and two additional, arbitrarily defined vectors **e**_*x*′_ and **e**_*y*′_ perpendicular to **e**_*z*′_. The transformation matrices **T**(*i*,*j*;*t*) at time *t* is then constructed with the unit vectors being columns **T** = (**e**_*x*′_, **e**_*y*′_, **e**_*z*′_). The pseudo forces in the Cartesian directions *x*, *y* and *z* (denoted with the Greek index *γ*) are then given by15
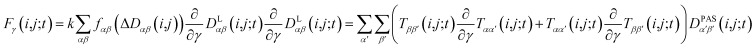



The calculation of the orientational pseudo forces is thus reduced to determining the derivatives of the elements of the transformation matrices **T**(*i*,*j*;*t*) with respect to the Cartesian coordinates of atoms *i* and *j* at time *t*. The direction of pseudo forces is calculated from the actual orientations of the dipolar *i*,*j*-system (*e.g.* a CH-vector), while the magnitude of pseudo forces depends on the difference between exponentially weighted a CH-vector), while the magnitude of pseudo forces depends on the difference between exponentially weighted 〈*D*L*αβ*(*i*,*j*))〉_*t*_ and the corresponding experimental dipolar coupling tensor components *D*exp*αβ*(*i*,*j*) as expressed by the function *f*_*αβ*_(Δ*D*_*αβ*_(*i*,*j*)).

### 2.6 Adapted pseudo force strength

In geometry optimization with NMR constraints, the standard harmonic form of the pseudo energy as given in eqn (13) is useful, especially if we are near the pseudo energy minimum. For the situation in MD simulations far from minima, however, the harmonic potential leads to rapidly growing forces and therefore to unrealistic structures and motions. These unrealistically large pseudo forces can be avoided by multiplying their values with a hyperbolic tangent weighting function, leading to16
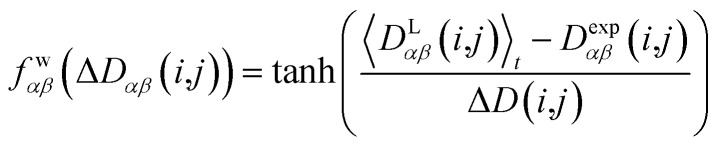



In this case, the width of the potential Δ*D*(*i*,*j*) is chosen ideally to be proportional to the estimated experimental error Δ*D*^exp^(*i*,*j*) derived from the coupling measurement, Δ*D*(*i*,*j*) ≈ Δ*D*^exp^(*i*,*j*). As long as the condition ). As long as the condition 〈*D*L*αβ*(*i*,*j*))〉_*t*_ – *D*exp*αβ*(*i*,*j*) < Δ*D*(*i*,*j*) is fulfilled, the function behaves similarly to the derivative of the original energy expression from eqn (13) divided by the experimental error, *i.e.* a linear dependence according to 
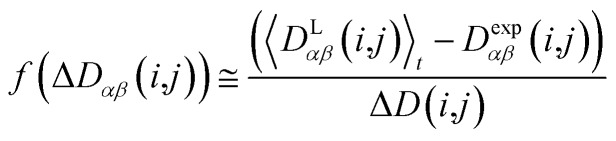
; but it gives rise to nearly constant forces if ; but it gives rise to nearly constant forces if 〈*D*L*αβ*(*i*,*j*))〉_*t*_ – *D*exp*αβ*(*i*,*j*) exceeds the threshold Δ*D*(*i*,*j*).^76^

The value of the pseudo force constant *k* is critical for the setup of successful MD simulations. Too big values may lead to unrealistic molecular motions, whereas too small values may result in insufficient sampling of the relevant conformations in reasonable computing times. A value of *k* = 10^–4^ kJ Hz^–2^ is usually a good starting compromise. The final pseudo force constants may vary between 10^–3^ and 10^–5^ kJ Hz^–2^, depending on the particular simulation. As long as the most favourable configurations do not exceed *χ*–2min values of 1 (eqn (21)), *k* should be increased to ensure proper sampling. Too high values for *k* can be identified by monitoring the temperature development during the course of an MDOC simulation: while a temperature rise can be tolerated as long as a dynamic equilibrium is reached, a steadily increasing temperature during the simulation is a clear sign of too large force constants.

Although dipolar couplings are downscaled with the scaling factor *s*_AM_, pseudo forces especially in the beginning of the MD simulation may become too strong. The molecules could surmount high barriers that separate configurations or form conformers which do not occur at ambient temperatures. Therefore an additional function is introduced that starts with zero and asymptotically reaches a value of one controlled by an additional time constant *ρ*, resulting in an overall weighting function of the form.

17 *f*_*αβ*_(Δ*D*_*αβ*_(*i*,*j*)) = (1 – e^–*t*/*ρ*^)*f*w*αβ*(Δ*D*_*αβ*_(*i*,*j*)).

Typical values for *ρ* are on the order of 200 ps, which lead to sufficient initial damping over the time course of the memory decay time constant *τ* of a similar duration.

### 2.7 Treatment of methyl groups

In the case of freely rotating X-CH_3_ groups^51^ the averaging of the dipolar couplings of these groups is taken into account by computing the value for a virtual CH (vCH) vector pointing along the X–C rotation axis and then applying the resulting forces on the methyl carbon and its α-substituent X using the relation18
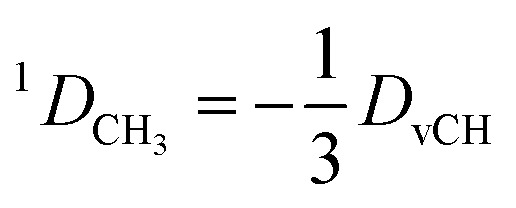



## 3 Materials and methods

### 3.1 NMR spectroscopy

**Fig. 1:**
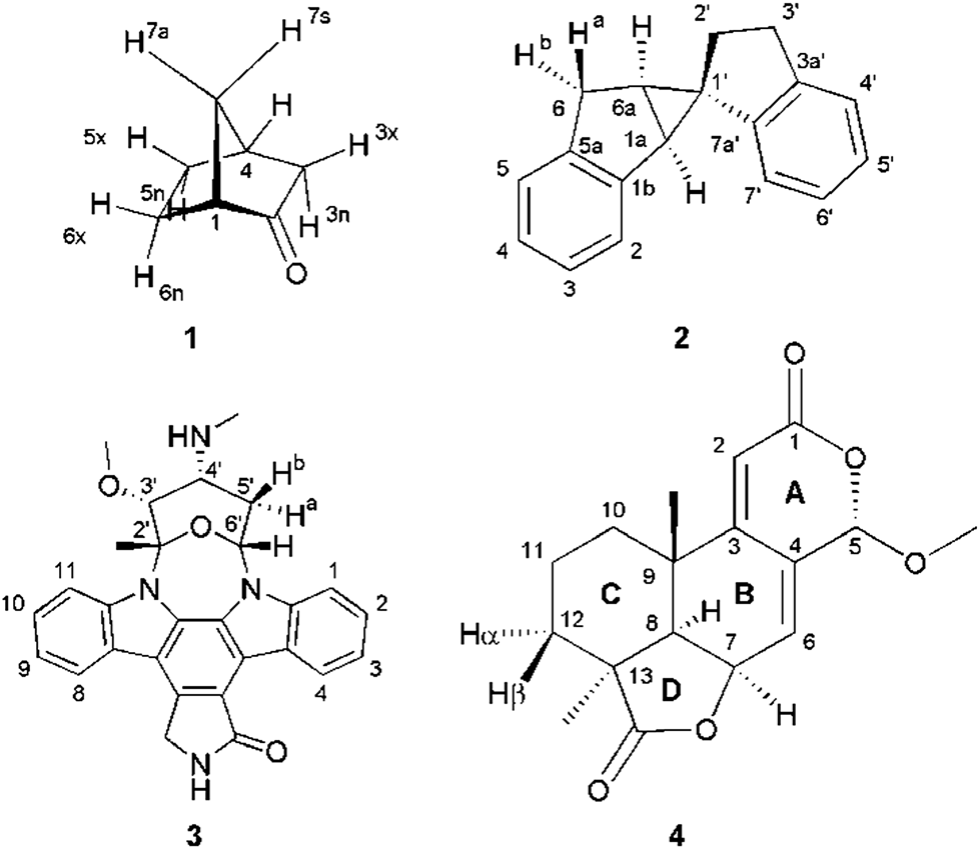
Structure and numbering of the four test molecules: norcamphor (**1**), a synthetic spiroindene derivative (**2**), staurosporine (**3**) and oidiolactone B (**4**).

Four example molecules with different properties have been chosen to demonstrate the capabilities of the MDOC simulations with respect to prochiral assignment, conformation, and determination of the relative configuration (see Fig. 1): norcamphor **1** and a synthetic spiroindene derivative **2** as rigid systems, staurosporine **3** as an example for a molecule with several potential conformations, and oidiolactone B **4** as a molecule with inherent flexibility on the NMR time-scale. The RDC data for compound **1** aligned in PEOMMA/TFE,^19^ compound **2** aligned in CDCl_3_/PDMS,^8^ and compound **3** aligned in dPS/CDCl_3_^10^ were taken from the literature. Oidiolactone B (**4**) was aligned in polyacrylonitrile/DMSO-*d*_6_ 16 using a rubber-based stretching device.^84^,^85^ The assignment of ^1^H and ^13^C chemical shifts as well as the measured proton–proton scalar couplings are provided as the ESI.† Note that the assignment and RDC data for oidiolactone B are given using the carbon numbering shown in Fig. 1. The known absolute stereochemistry of oidiolactone B is 5*S*7*R*8*R*9*S*13*S*. All dipolar couplings are defined as the difference *D* = *T* – *J* between the measured splittings under aligned and isotropic conditions. Both ^1^*T*_CH_ and ^1^*J*_CH_ couplings for **4** have been obtained from CLIP-HSQC experiments^86^ in a stretched gel and in pure isotropic DMSO-*d*_6_, respectively.

### 3.2 Molecular dynamics simulations

MDOC simulations were performed using the COSMOS 6.0 implementation of the COSMOS-NMR force field.^87^,^88^ Atomic partial charges are recomputed every 2 fs by using bond polarization theory (BPT).^89^,^90^ For the more rigid systems norcamphor **1** and spiroindene **2**, 10 ns MDOC simulations were employed whereas for the more flexible staurosporine **3** and oidiolactone B **4** longer MDOC simulations of 20 ns were necessary to reach equilibrium. One bond C–H distances in norcamphor and spiroindene were fixed using the SHAKE algorithm,^91^ which allowed an integration time step of up to 2 fs. A memory decay time constant *τ* of 200 ps was employed in all cases. A force reaction time *ρ* for the uprising of the pseudo forces was set to 200 ps. The optimal values for the pseudo force constant *k* and the scaling factor *s*_AM_ change from system to system and have been subjected to optimization. In the case of the force constant *k* we increased its value successively until the best configurations fulfilled experimental constraints within errors and checked at random that only physically meaningful structures were obtained. Also the optimization of the scaling factor *s*_AM_ is crucial for the outcome of the simulations as it determines the maximum difference Δ*D*_*αβ*_ at any given time *t*. A large factor *s*_AM_ will increase the effective pseudo forces similar to the force constant *k*, so the interdependence of the two factors must be taken into account. But even if the product *k s*_AM_ is kept constant, the size of the scaling factor has a strong influence on the populated conformations. If *s*_AM_ is too large, Δ*D*_*αβ*_ can be on the order of kHz, so that small deviations of RDCs of a few Hz are easily compensated by very small angular changes of *e.g.* 1° and below. As a result, essentially a single major conformation is populated with only slight vibration-like variations in individual bonds that cannot fulfil all experimental constraints. Too small factors *s*_AM_ < *s*expAM, on the other hand, have a similar effect as the largest experimental constraints can never be attained and the corresponding bond vector will essentially be fixed along the *z*-axis. The best results are obtained for scaling factors on the order of 2–10 times *s*expAM, where the latter can be estimated by the largest component of an approximate Saupe matrix *S*_*αβ*_, as mentioned above. As a rule of thumb, also the largest absolute value of experimental RDCs can be used as a good initial estimate: the largest values of approximately 10, 20, and 30 Hz showed good performance with *s*_AM_ values of 0.001, 0.002, and 0.003, respectively.

The simulations were performed in the NVT ensemble with the temperature typically being set to 300 K and controlled *via* a proper thermostat.^92^

### 3.3 Data interpretation

All reported averaged properties are computed by discarding the first nanosecond of the MD simulations. The fit of computed dipolar couplings to the experimental ones is expressed by two overall quality criteria that turn out to be very useful for the evaluation of how far the structural models are consistent with the experimental data. Both criteria are based on *χ*^2^, a well-known quantity that reliably describes the difference of calculated *vs.* experimental data with respect to the error of the experiment. For each coupling between spins *i* and *j*, a corresponding individual criterion can be formulated according to19
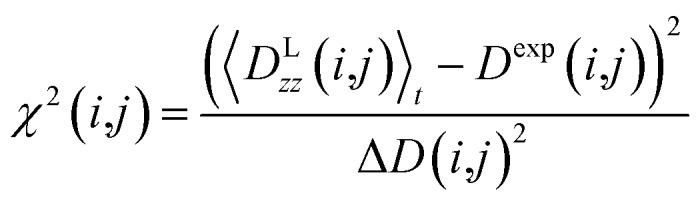
where the calculated RDC is represented by the *zz*-component of the weighted dipolar tensors -component of the weighted dipolar tensors 〈**D**^L^(*i*,*j*))〉_*t*_ as calculated from the MDOC trajectory (see eqn (8) and (10), respectively) or by any other theoretically derived dipolar coupling as for example calculated by the program MSpin using a single alignment tensor fit based on singular value decomposition. In the case of MDOC, typically the first nanosecond of the trajectory is discarded as large initial structural fluctuations in the molecular dynamics simulation are unavoidable and of no relevance to the subsequent data interpretation. It should be noted that the criterion could also easily be based on the differences between all nine tensorial components of , respectively) or by any other theoretically derived dipolar coupling as for example calculated by the program MSpin using a single alignment tensor fit based on singular value decomposition. In the case of MDOC, typically the first nanosecond of the trajectory is discarded as large initial structural fluctuations in the molecular dynamics simulation are unavoidable and of no relevance to the subsequent data interpretation. It should be noted that the criterion could also easily be based on the differences between all nine tensorial components of 〈**D**^L^(*i*,*j*))〉_*t*_ and **D**^exp^(*i*,*j*), but we found empirically that the *zz*-component is clearly representative of all tensor components and ensures a fair comparability between the different approaches.

The first overall quality criterion we use to describe the overall fit with the experimental data is the inverse normalized *χ*^2^ factor^27^20
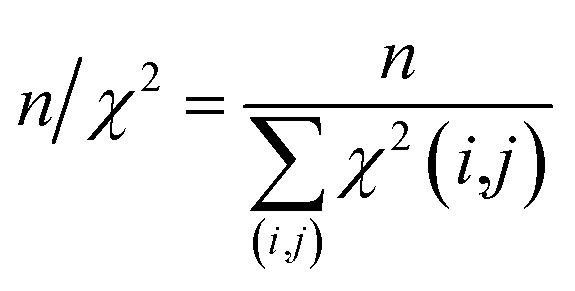
where the sum runs over the *n* spin pairs (*i*,*j*) for which experimental data are available. If *n*/*χ*^2^ is smaller than 1, it clearly indicates that the theoretical model is not fully consistent with the experimental data within the experimental error. It serves therefore as a first criterion to sort out structural models that do not comply with all experimental constraints. On the other hand, a value larger than 1 does not necessarily indicate consistency, as *n*/*χ*^2^ ≥ 1 represents a necessary, but not a sufficient, condition. We therefore also introduce here an even tighter overall criterion, which is the minimum of the reciprocal individual *χ*^2^(*i*,*j*) values21
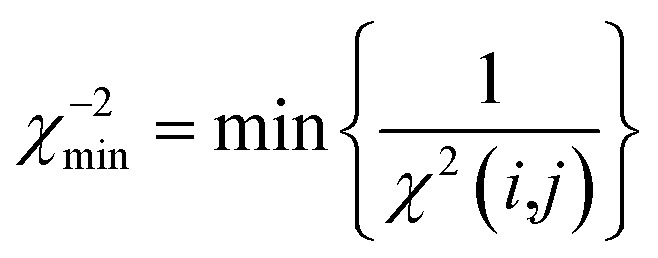



Only if this criterion is equal to or greater than 1, the structure fulfils all experimental data within the given error. We also give the number of outliers, *i.e.* the number of spin pairs with 1/*χ*^2^(*i*,*j*) < 1 that violate the structure, as an additional parameter for the evaluation of a particular structure within parentheses.

## 4 Results and discussion

In the current work we have evaluated the performance and capabilities of molecular dynamics with orientational constraints (MDOC) simulations as implemented in COSMOS 6.0 to assign the relative configuration of stereogenic and prochiral centres in small organic molecules. We have selected a set of four compounds with varying degrees of conformational mobility, starting from a rigid skeleton through semi-rigid cases to fully flexible systems in fast exchange (Fig. 1). In the following, the MDOC setup and results for the different cases are given in detail and discussed, including the corresponding standard alignment tensor analyses.

### 4.1 Conformationally rigid systems: norcamphor

The bridged ring system of norcamphor **1** is an example molecule with an assumedly rigid skeleton. All possible ^1^*D*_CH_ values of **1** in a PEOMMA/TFE gel have been measured using a CLIP-HSQC experiment^19^,^86^ (see Table 1). Thus previously reported experimental RDCs define a complete set of independent RDCs for a thorough RDC data evaluation.

**Table 1:** Experimental *vs.* computed one-bond RDCs for norcamphor

Sites	Experiment	Expt. Err.	MDOC	SVD
	*D* ^exp^ ^*a*^/Hz	Δ*D*^*b*^/Hz	〈*D*L*zz*〉_*t*_^*c*^/Hz	*D* ^calc^ ^*d*^/Hz
C_1_H_1_	1.4	0.5	1.4	0.8
C_3_H_3x_	6.0	0.7	5.6	5.9
C_3_H_3n_	5.0	0.7	4.6	5.8
C_4_H_4_	–2.9	0.3	–2.8	–2.3
C_5_H_5x_	–10.5	0.5	–10.0	–10.6
C_5_H_5*n*_	5.3	0.5	5.3	5.0
C_6_H_6x_	–1.9	0.5	–2.1	–1.9
C_6_H_6n_	1.5	0.2	1.4	1.0
C_7_H_7s_	5.8	0.2	5.7	5.8
C_7_H_7a_	–7.1	0.2	–6.9	–7.0

^*a*^Experimental values for ^1^*D*_CH_ residual dipolar couplings.

^*b*^Corresponding experimental maximum error estimates,^93^ corresponding to roughly three times the standard deviation.

^*c*^Time averaged computed RDCs from the MDOC trajectory using the correct assignment.

^*d*^Back calculated RDCs using the SVD-based alignment tensor approach implemented in the program MSpin^52^ and the correct assignment.

The conformationally stable structure of norcamphor allows to establish a basic protocol and to evaluate the performance of the method without significant complications and ambiguities arising from conformational averaging of the RDC data. It will also allow the determination of the influence of vibrational averaging and how far MDOC computations can be used to create an ensemble of conformers that fulfil the experimental data. Norcamphor has ten C–H vectors, where eight are part of four methylene groups, out of which three are having diastereotopic *endo*-/*exo*-positions and one a *syn*-/*anti*-position (Fig. 1, Table 1). The assignment of the *endo*-/*exo*- and *syn*-/*anti*-positions can be unambiguously achieved based on scalar coupling and NOE data. However, for the evaluation of MDOC simulations, we also have performed the prochiral assignment by means of analysis *via*^1^*D*_CH_ RDCs.

As a first step, a starting geometry of **1** was optimized at the AM1 level of theory,^94^ from which an input file for COSMOS was built containing the molecular geometry and connectivities, along with atom definitions, dipolar coupling data and control keywords (see the ESI†). The aliphatic C–H bond distances were fixed to 1.093 Å, the default value for the corresponding tensorial COSMOS force field, using the SHAKE algorithm.^91^ Additionally, all distances from each methylene proton to the respective β-carbons and the methylene interproton distances were fixed during the MDOC simulation.

We then started MDOC simulations with ten tensorial constraints from the experimental ^1^*D*_CH_ couplings, varying the pseudo force strength *k* and the scaling factor *s*_AM_. Good conditions, as judged by the introduced quality criteria and the equilibration of the trajectory, were obtained for *k* = 6 × 10^–4^ kJ Hz^–2^ and *s*_AM_ = 10^–3^.

**Fig. 2:**
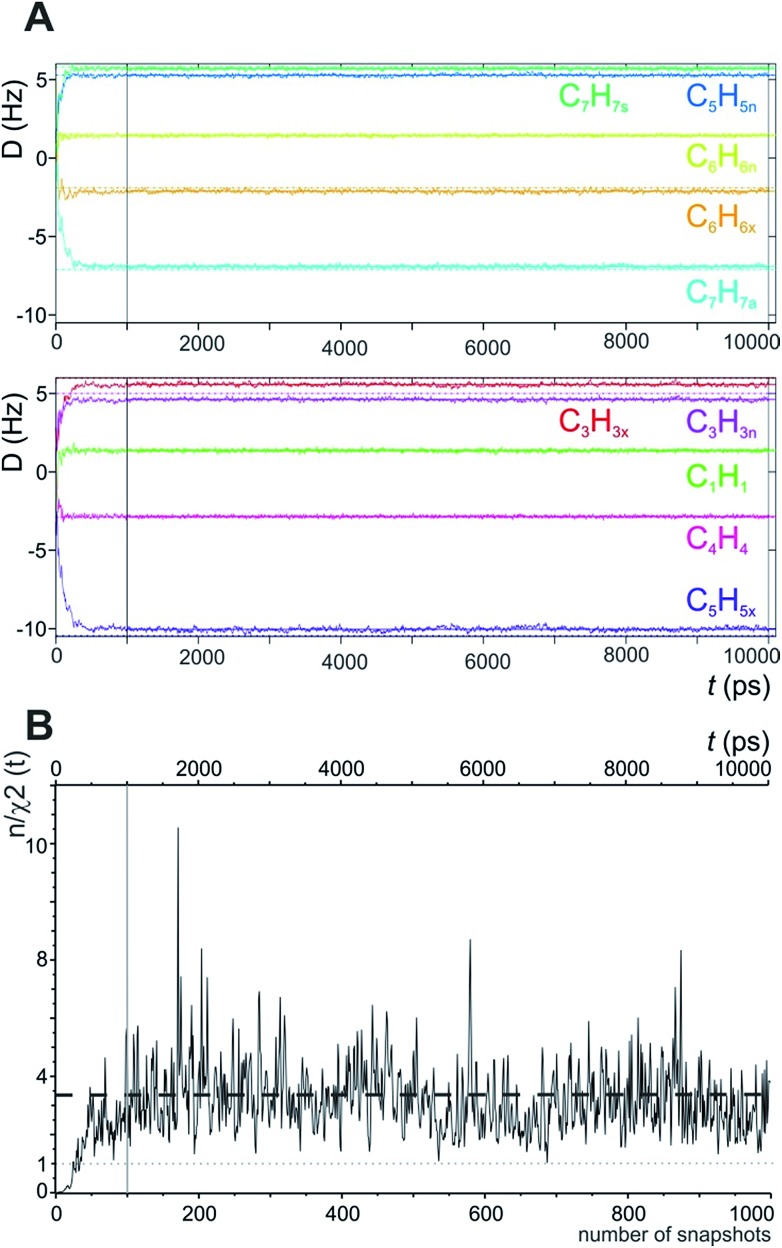
(A) Time course of individual simulated RDC values during the 10 ns MDOC simulation of norcamphor. The individual RDCs are assigned and colour coded with the experimental values given as horizontal dotted lines. Shortly after the start of the MDOC simulation, *i.e.* after approximately 250 ps, all ^1^*D*_CH_ values fluctuate within or near the corresponding error ranges. (B) Evolution of the quality criterion *n*/*χ*^2^ over the MDOC simulation of norcamphor using the correct assignment: After 500 ps for every snapshot with weighted time averaging according to eqn (10), *n*/*χ*^2^ is always larger than 1 (grey dotted line). The dashed horizontal line shows the final value of the quality criterion (3.314). The first nanosecond is needed for building up the correct orientational and structural averaging and is left out from the final analysis (vertical solid lines in A and B).

In the MDOC simulation with the correct prochiral assignment, all the computed dipolar couplings converged to the experimental values within the error limits (see Table 1). A closer look at the time evolution of the corresponding *D*L*zz*(*i*,*j*) values reveals that convergence is reached in a time interval of approximately 250 ps, *i.e.* slightly longer than the time period *ρ* = 200 ps for the uprising of the pseudo forces (Fig. 2A). After convergence is reached, only small fluctuations of the computed RDC values are visible. The effect can also be seen in the overall quality criterion *n*/*χ*^2^ over the course of the trajectory as shown in Fig. 2B. A plateau value for the quality criterion above 1 is found after *ca.* 150 ps of the MD simulations, indicating averaged orientations that potentially fulfil the experimental constraints as soon as the corresponding pseudo forces are active. To be on the safe side, we decided in the following to just use converged time steps for subsequent structure analysis by discarding the first nanosecond of the trajectory. The resulting averaged plateau value for *n*/*χ*^2^ is 3.314 (Table 2 and the dashed line in Fig. 2B).

**Table 2:** Assignment of diastereotopic methylene protons in norcamphor

Swap^*a*^	MDOC		SVD	
	*n*/*χ*^2^^*b*^	*χ* –2 min ^*c*^	*n*/*χ*^2^^*d*^	*χ* –2 min ^*e*^
	3.314	1.160 (0)	0.671	0.137 (4)
X3	2.700	0.828 (1)	1.017	0.207 (3)
X5	0.017	0.002 (7)	0.008	0.003 (10)
X6	0.937	0.119 (1)	0.190	0.034 (6)
X7	0.004	0.001 (5)	0.005	0.001 (8)

^*a*^Carbon position at which the assignment of two methylene protons is exchanged relative to the correct assignment.

^*b*^Initial overall quality criterion applied to the MDOC simulations.

^*c*^Strict overall quality criterion for the MDOC run, the number of outliers is given within brackets.

^*d*^Corresponding overall quality criterion computed from the MSpin alignment tensor results.

^*e*^Corresponding strict overall criterion for MSpin results.

For a first demonstration of the capabilities of MDOC in structural analysis, we had a closer look at the potential to determine the prochiral assignment within methylene groups. Ample examples have been reported previously that prochiral assignments can be achieved using ^1^*D*_CH_ couplings in alignment tensor calculations as long as the two methylene RDCs differ sufficiently.^95^,^96^ A straightforward method is the comparison of quality criteria for the two possibilities of the prochiral assignment. There are four methylene groups in norcamphor, so we constructed four additional data sets by swapping the two ^1^*D*_CH_ values of a single methylene group in each set. We refer to the data sets as X3, X5, X6, and X7 for the C3, C5, C6, and C7 prochiral carbons, for which the assignment of attached protons has been exchanged. An MDOC simulation was performed for each of these data sets using the already optimized parameters. As can be seen in Table 2, only the MDOC simulations corresponding to the correct assignment and the swapping at position C3 (X3) fulfil the quality criterion *n*/*χ*^2^ > 1, whereas in all other cases the prochiral assignment can be excluded. The weak discrimination in the case of the C3 carbon, on the other hand, is expected since the two ^1^*D*_CH_ couplings (C_3_H_3x_ and C_3_H_3n_) present close values. The strict *χ*–2min criterion exhibits a slightly improved discrimination as it reveals a better fit of the correct structure while one outlier is detected for the X3 assignment (see Tables 1 and 2).

For a fair comparison with state-of-the-art techniques, the different data sets were also fitted to a single MMFF94 (ref. 97) optimized geometry using the well-established SVD-based alignment tensor approach as implemented in MSpin.^52^ The averaged back calculated RDCs for the correct assignment are listed in Table 1 next to the MDOC-derived RDCs. An *n*/*χ*^2^ value of 0.671 from the back calculated values and a condition number in the SVD decomposition of 2.72 are obtained, indicating that the order tensor could be confidently calculated from the data set. The MSpin-fitted data generally correlate very well with the experimental data, but a closer look also reveals that four RDCs are clearly outside the error range of the experiment. The outliers imply that the structural model does not completely fulfil the experimental data. Apparently, the experimental RDC values cannot be obtained with the computed conformation, which leads to the conclusion that either the static structural model on the MMFF94 level is incorrect, or, more likely, the significant deviations result from missing vibrational averaging and are therefore inherent to the static method. Regarding the determination of the prochiral assignment, the alignment tensor approach implemented in MSpin equally works in discarding the assignments X5, X6, and X7. Interestingly, the *n*/*χ*^2^ and *χ*–2min values slightly favour the wrong assignment X3 (1.067 and 0.207 using MSpin) over the correct assignment (0.671 and 0.137, respectively).

### 4.2 Conformationally rigid systems: a spiroindene derivative

**Fig. 3:**
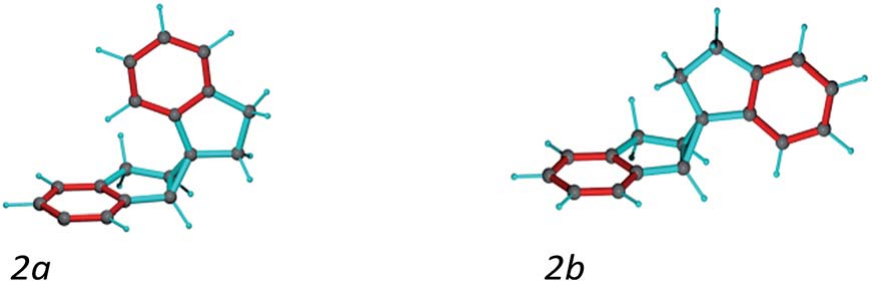
Spiroindene with the incorrect (**2a**) and correct (**2b**) relative configurations used in the MDOC simulations.

A second test on the ability of the COSMOS implementation of MDOC to discriminate between diastereoisomeric structures is performed on spiroindene **2** (Fig. 1). The configuration of the molecule, according to X-ray analysis,^98^ has been determined to be 1*S*1a*S*6a*R*, which corresponds to configuration **2b** (Fig. 3). A previous RDC analysis of **2** using ^1^*D*_CH_ couplings obtained in a stretched cross linked poly(dimethylsiloxane)/CDCl_3_ gel resulted in data that could be fitted well using the SVD-fitting procedure as well as the steric prediction method implemented in PALES.^45^ A good discrimination between **2a** (1*R*1a*S*6a*R*) and its diastereoisomer **2b** was observed.^85^ In the following we use this well-studied test case to evaluate the ability of the MDOC simulations to discriminate the diastereoisomers **2a** and **2b**.

As in the norcamphor case, the distances between methylene protons and their respective β-carbons were fixed with the SHAKE algorithm. Equally, all C–H vectors were fixed to the COSMOS standard distances for ^1^*D*_CH_ couplings. The first MDOC test runs showed that the influence of the orientational pseudo-forces on the tetrahedral geometry of the quaternary carbon in the cyclopropyl ring cannot be compensated by the standard force field implemented in COSMOS. As a result, unphysically flattened structures appeared, followed by a drastic increase in temperature and premature ending of the MDOC simulations without convergence. To circumvent this problem and preserve the tetrahedral geometry, we fixed the distances between the pairs of carbons next to the quaternary spiro-carbon using SHAKE. With these additional distance constraints, the geometries of both **2a** and **2b** were well maintained during all MDOC simulations. Optimization of the pseudo forces and scaling factor led to *k* = 4.5 × 10^–5^ kJ Hz^–2^ and *s*_AM_ = 2 × 10^–3^, which were used for the final MDOC simulations of the two possible relative configurations.

The spiroindene experimental data as shown in Table 3 contain RDC values within a range of 10 Hz (–3.5 to 6.4 Hz) and relatively large experimental errors (1.0 to 2.0 Hz). Since the individual experimental error values are used within the MDOC simulations to determine the width of the potentials for the constraints, it could be expected that the discrimination of the diastereoisomers will be less pronounced as with the alignment tensor approach using a single rigid structure. Nevertheless, as can be seen in Table 3, the averaged calculated RDC values during the MDOC simulation for the correct diastereoisomer very well reproduce the experimental ones and the comparison of the final *n*/*χ*^2^ values of 0.268 for **2a** and 11.280 for **2b** allows a clear differentiation between the diastereomers and the presence of 7 outliers for **2a** and no outliers for **2b** leave no doubt about the correct relative configuration. For completeness, we repeated a previously published SVD-based alignment tensor analysis with the program MSpin,^52^ which essentially led to identical results as previously published. It should be noted that the impact of the vibrational averaging in the analysis can safely be neglected as the large relative experimental errors of up to 30% in the experimental data render it irrelevant (Table 4).

**Table 3:** Experimental and computed data for spiroindene **2b**

Sites	Experiment		MDOC	SVD
	*D* ^exp^ ^*a*^/Hz	Δ*D*^*b*^/Hz	〈*D*L*zz*〉_*t*_^*c*^/Hz	*D* ^calc^ ^*d*^/Hz
C_2_H_2_	–2.2	1.0	–1.8	–2.2
C_3_H_3_	–0.3	1.0	–0.2	0.2
C_4_H_4_	4.9	1.0	4.9	5.5
C_5_H_5_	–2.1	2.0	–1.8	–2.3
C_1a_H_1a_	–2.0	2.0	–2.1	–2.0
C_6_H_a6_	–1.9	1.0	–2.3	–2.1
C_6_H_b6_	–3.5	1.0	–3.0	–3.0
C_6a_H_6a_	–0.2	2.0	–0.2	–1.4
C_4′_H_4′_	–1.7	1.0	–1.3	–1.4
C_5′_H_5′_	6.4	1.5	5.8	5.6
C_6′_H_6′_	–1.3	1.0	–0.8	–0.4
C_7′_H_7′_	–1.1	2.0	–1.3	–1.4

^*a*^Experimental values for ^1^*D*_CH_ residual dipolar couplings.

^*b*^Corresponding experimental error estimates.^93^

^*c*^Time averaged computed RDCs from the MDOC trajectory using the correct assignment.

^*d*^Back calculated RDCs using the SVD-based alignment tensor approach implemented in the program MSpin^52^ and the correct assignment.

**Table 4:** Configurational assignment for spiroindene

Config.	MDOC		SVD	
	*n*/*χ*^2^^*a*^	*χ* –2 min ^*b*^	*n*/*χ*^2^^*c*^	*χ* –2 min ^*d*^
**2a**	0.268	0.047 (7)	0.178	0.035 (8)
**2b**	11.280	4.623 (0)	5.276	1.678 (0)

^*a*^Overall MDOC quality criterion.

^*b*^Strict overall quality criterion for the MDOC simulation; the number of outliers is given within brackets.

^*c*^Same overall quality criterion computed from the MSpin alignment tensor results.

^*d*^Same strict overall criterion for MSpin results.

### 4.3 Conformational variability: staurosporine

The next well-studied model molecule, this time with four stereogenic centres and conformational freedom in a six-membered ring, is staurosporine **3** (Fig. 1). Staurosporine is a protein kinase inhibitor isolated from *Streptomyces staurosporeus*. Its absolute configuration has been determined by single crystal X-ray analysis of its *N*-methyl iodide derivative to be 2′*S*3′*R*4′*R*6′*R*.^99^^1^*D*_CH_ RDCs have been measured for staurosporine aligned in stretched perdeuterated polystyrene/CDCl_3_ gels.^10^ In its analysis the authors have shown that next to the stereochemistry two possible conformational states for the six-membered ring, chair and boat, have to be considered as well. An SVD-based fit of RDCs showed the best correlation with the chair conformation.^10^

For the MDOC simulations, the geometry of the correct configuration was initially optimized using the MM2 force field (Chemdraw 3D, Cambridge Software). Then, as in the previous examples, the distances between methylene protons and their respective β-carbons were fixed with the SHAKE algorithm and all C–H vectors were fixed to the COSMOS standard distances for ^1^*D*_CH_ couplings. The pseudo force constant and the scaling factor were optimized for the molecule to *k* = 2 × 10^–3^ kJ Hz^–2^ and *s*_AM_ = 1 × 10^–3^, respectively. For a conformational search and for input in the SVD-based alignment tensor approach in MSpin, all low-energy geometries for the different combinations of configurations and conformations were optimized at the DFT BP86 level.

**Fig. 4:**
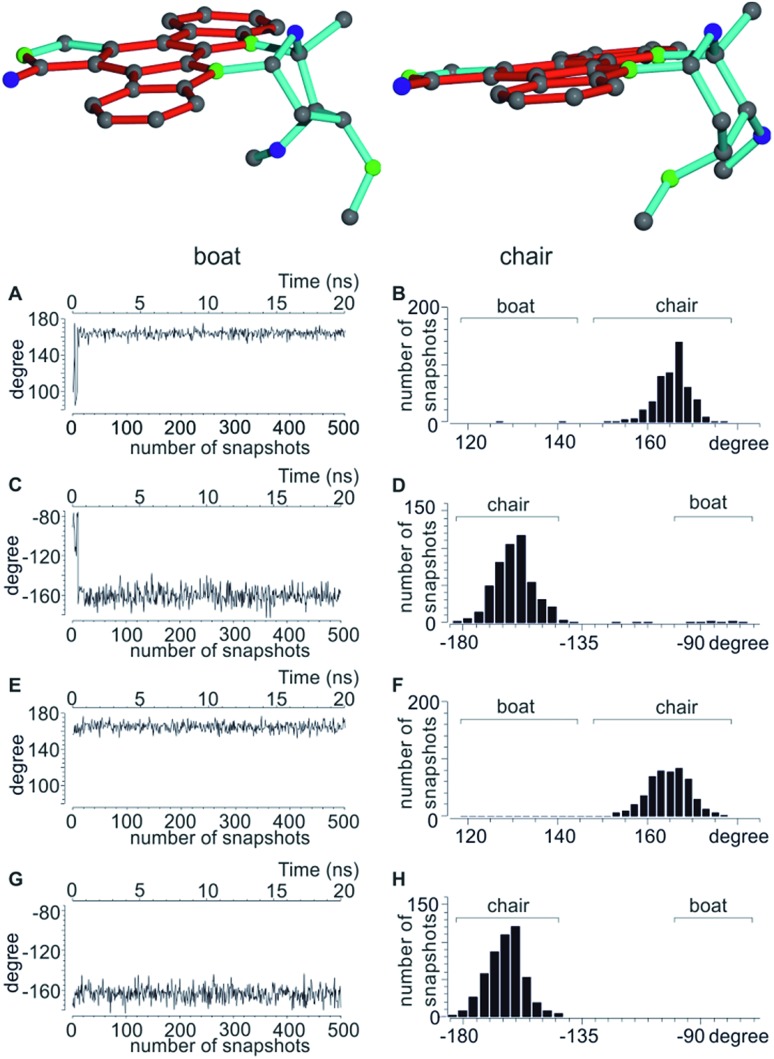
Top: boat and chair conformers of staurosporine. Bottom: analysis of the dihedral angles CH_3_-C2′-C3′-C6′ and H6′-C6′-C5′-C4′ of the 2′*S*3′*R*4′*R*6′*R* configuration indicative of the two possible conformations. (A–D) dihedral angles over the initial part of the MDOC trajectory starting with the *SRRR*b structure (A and C) with the corresponding angular distribution over the whole MDOC simulation (B and D). After a short initial period the conformation switches from boat to chair, where it stays for the majority of the MDOC simulation. (E–H) Corresponding dihedral angles during the same part of the trajectory starting with the *SRRR*c conformation (E and G) and the overall angular distribution (F and H).

In accordance to previous RDC analysis we evaluated all possible relative configurations on the 2′, 3′, 4′ and 6′ stereogenic centres taking into account both conformations (chair and boat) in every case. Using the program Avogadro, all structural isomers were produced and geometry optimized. For visualization, the boat and chair conformations for the correct configuration are shown in Fig. 4. As the nomenclature for the various configurations of **3**, a simple stereogenic descriptor in the order of the numbering is given, such that the *SRRR* configuration of the six-membered ring corresponds to the correct configuration 2′*S*3′*R*4′*R*6′*R*. The conformation of the six-membered ring is abbreviated with either b for boat or c for chair.

As shown in Table 5, both MDOC and MSpin derived RDCs correlate well with the experiment. MSpin, using rigid structures and essentially five RDCs for defining the six-membered ring with the four stereogenic centres as input, can clearly distinguish *SRRR*c as the best structural model. *SRSR*c as the second best fitting structure still achieves an *n*/*χ*^2^ > 1, but it shows also three outliers as compared to only one for the correct structure and can be excluded therefore (Table 6). Again, most likely due to missing vibrational averaging in the static approach, even the correct structure cannot fully represent all RDCs within their relatively large experimental errors. Using MDOC, the five one-bond RDCs with relatively large error ranges are barely sufficient to define the flexible ring. They leave a large conformationally unrestricted space that will be entirely used to fulfil the averaged RDCs. Considering this freedom in conformational space, we were positively surprised that the MDOC simulations still show a fair discrimination capability between the trial configurations. The correct configuration and chair conformation *SRRR*c has a total *n*/*χ*^2^ value of 4.139, and the closest competitor, the *SRSR* stereoisomer with the boat conformation, has a total *n*/*χ*^2^ of 2.657, not allowing any discrimination *per se*. However, only the correct structure fulfils all constraints, while *SRSR*b displays a *χ*–2min of 0.481, *i.e.* not all experimental RDCs are fulfilled within the error range. Equally, for all other structures at least one outlier is observed and the corresponding *χ*–2min values are even smaller. It can be concluded that despite the much larger molecular flexibility inherent to the MDOC approach compared to the discrete conformational SVD-based fit, the correct configuration can still be distinguished from the incorrect ones.

**Table 5:** Staurosporine RDC data for the 2′*S*3′*R*4′*R*6′*R* configuration in the chair conformation (*SRRR*c)

Sites	Experiment		MDOC	SVD
	*D* ^exp^ ^*a*^/Hz	Δ*D*^*b*^/Hz	〈*D*L*zz*〉_*t*_^*c*^/Hz	*D* ^calc^ ^*d*^/Hz
C_2′_CH_3_	–3.2	1.0	–2.7	–2.7
C_3′_H_3′_	–19.8	5.0	–15.0	–20.8
C_4′_H_4′_	–3.7	1.0	–3.7	–4.2
C_5′_H_5′a_	7.5	3.2	7.4	7.4
C_6′_H_6′_	5.8	1.1	5.1	5.7
C_1_H_1_	–27.2	2.5	–25.8	–27.8
C_2_H_2_	–11.1	7.6	–8.4	–10.6
C_3_H_3_	1.7	4.2	1.2	0.6
C_4_H_4_	–27.1	1.0	–26.5	–27.1
C_8_H_8_	–25.3	1.0	–24.8	–25.1
C_9_H_9_	–12.9	5.4	–10.9	–13.5
C_10_H_10_	2.6	3.6	2.2	2.6
C_11_H_11_	–25.4	1.1	–24.8	–24.0

^*a*^Experimental values for ^1^*D*_CH_ residual dipolar couplings.

^*b*^Corresponding experimental maximum error estimates derived as described in ref. 93.

^*c*^Time averaged computed RDCs from the MDOC trajectory using the correct configuration and chair conformation.

^*d*^Back calculated RDCs using the SVD-based alignment tensor approach implemented in the program MSpin using the correct configuration and chair conformation.

**Table 6:** Evaluation of the quality criteria for staurosporine

Config.^*a*^	MDOC		SVD	
	*n*/*χ*^2^^*b*^	*χ* –2 min ^*c*^	*n*/*χ*^2^^*d*^	*χ* –2 min ^*e*^
*SRRR*b	3.344^*f*^	0.905 (1)^*f*^	0.108	0.014 (8)
*SRRR*c	4.139	1.074 (0)	5.392	0.617 (1)
*SRSR*b	2.657^*f*^	0.481 (1)^*f*^	0.049	0.004 (5)
*SRSR*c	2.469	0.446 (1)	1.377	0.160 (3)
*SSRR*b	2.454^*f*^	0.374 (1)^*f*^	0.032	0.005 (10)
*SSRR*c	1.573	0.184 (2)	0.118	0.014 (7)
*SSSR*b	1.601^*f*^	0.220 (2)^*f*^	0.116	0.017 (8)
*SSSR*c	1.575	0.219 (2)	0.085	0.023 (10)

^*a*^Indicates the different configurations, where the order of the labelling for the stereogenic centres is kept according to 2′, 3′, 4′ and 6′; b and c indicate boat or chair starting conformations of the flexible ring.

^*b*^Overall MDOC quality criterion.

^*c*^Strict overall quality criterion for the MDOC simulation; the number of outliers is given within brackets.

^*d*^Same overall quality criterion computed from the MSpin alignment tensor results.

^*e*^Same strict criterion for MSpin results.

^*f*^Boat conformation changes to mainly chair conformation during the MDOC simulation.

A significant difference of the MDOC simulations compared to the MSpin single structure fits is the lack of distinction of different starting conformers, as all stereoisomers show very similar overall quality criteria for the two different starting conformers. The reason becomes clear when we look at Fig. 4, where two torsion angles representative of the boat and chair conformations are observed over the course of the trajectory for the *SRRR*b and *SRRR*c starting structures: after less than 1 ns the boat conformation flips into the chair conformation, where it stays for most of the rest of the simulation. Because of the change into the preferred chair conformation, the MDOC simulation with the *SRRR*b starting structure is practically indistinguishable from the one starting with *SRRR*c, as the MDOC calculations for the two starting conformations evaluated in Table 6 essentially contain the same conformational distribution as long as the initial 1 ns is discarded. It is therefore a good example for demonstrating that the MDOC procedure can induce strong enough pseudo forces to overcome the relatively high energy barriers associated with ring pseudo rotations, and thus to drive the molecule into its preferred conformations that best fulfil RDC constraints. Similar to the configuration shown, the starting boat conformations of the other stereoisomers always undergo a transition to the preferred chair conformations in the corresponding MDOC simulations.

### 4.4 Conformational flexibility: oidolactone B

A real life example for the MDOC analysis with an apparent degree of flexibility is oidiolactone B (**4**). The mould isolated compound^100^,^101^ shows activity against human fungal infections and cancer cell lines.^102^ The absolute configuration of its five stereogenic centres has been established as 5*S*7*R*8*R*9*S*13*S* (Fig. 1) *via* total synthesis and X-ray analysis.^103^ In the following, configurations are defined by the stereogenic descriptor in the order of the numbering, *i.e.* the correct configuration is given as *SRRSS*. It should be noted that methylene protons of **4** are labelled α, when they are situated behind the plain, while β labels indicate protons in front of the paper plain as shown in Fig. 1.

**Fig. 5:**
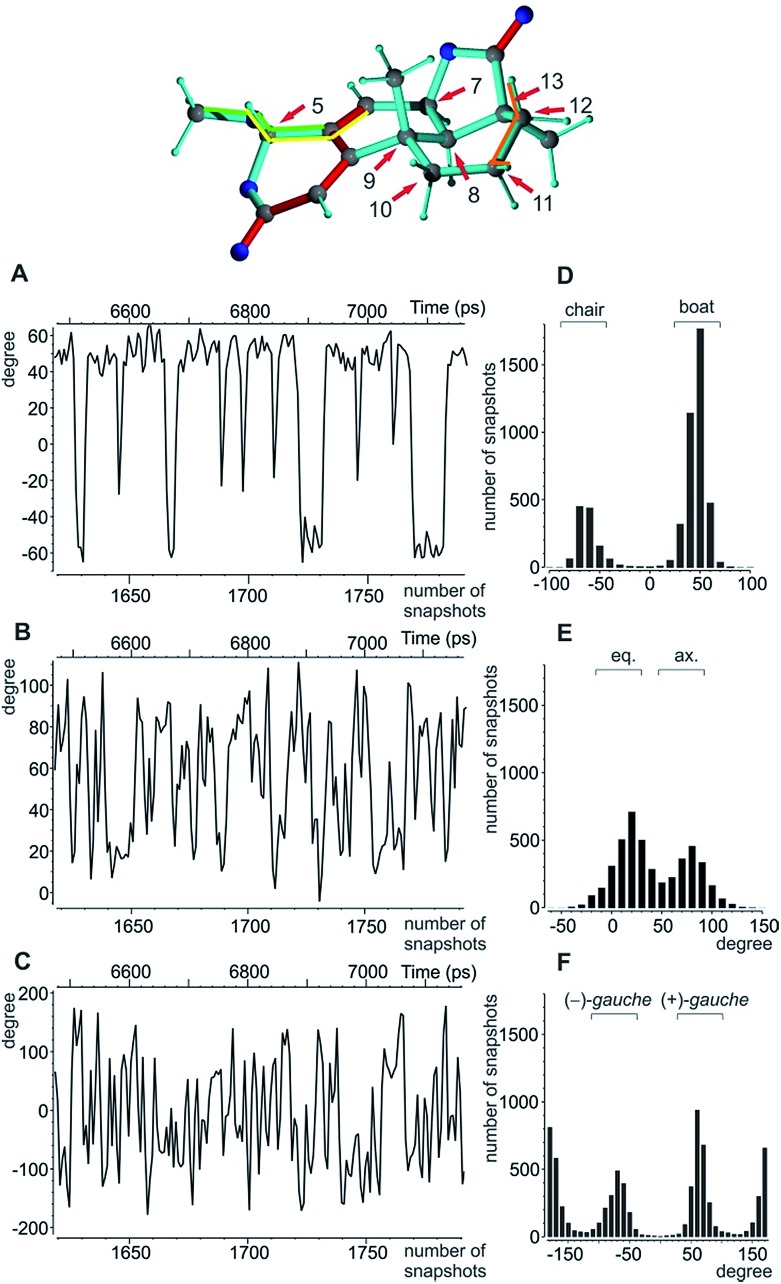
Analysis of the torsion angles for oidiolactone B. Top: boat conformation of oidiolactone B with red arrows marking the stereogenic and prochiral centres. The dihedral angles used to indicate conformational flexibility below are highlighted in orange, yellow, and green. (A) The dihedral angle H_11β_-C_11_-C_12_-H_12β_ as an indicator for the boat/chair conformation in ring C. A representative 650 ps excerpt of the trajectory is given. (B) The corresponding excerpt for the torsion angle O-C5-C4-C6 indicative of the axial/equatorial position of the methoxy group in ring A, and (C) the excerpt for the rotation of the methoxy group according to the dihedral angle C4-C5-O-CH_3_ are shown. The overall angular distributions for the angles in (A)–(C) are summarized in (D)–(F).

**Fig. 6:**
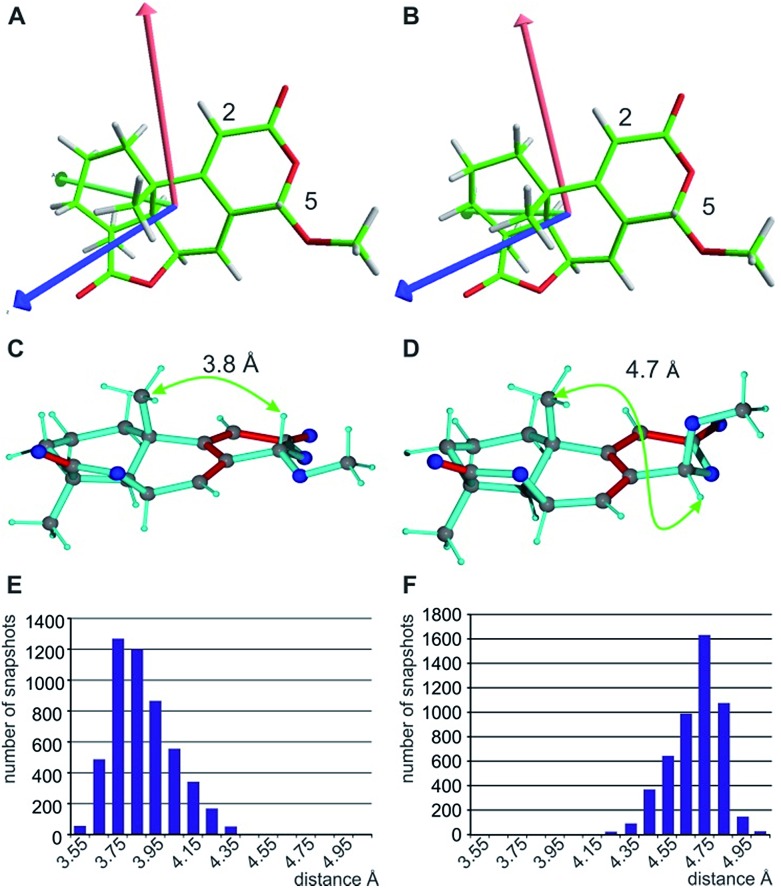
Alignment tensor consideration (A and B) and exclusion of the C5-epimer based on the NOE-derived distance (C–F). The fitted alignment tensors for the two major individual conformers with boat (A) and chair (B) arrangement in ring C are very similar (A_*xx*_ red, A_*yy*_ green, and A_*zz*_ blue), explaining the good applicability of the single tensor fit. The principle axes of the tensor are already predefined by the fixed orientation of the C5-H5 and C2-H2 vectors. The NOE-derived distance of approximately 3.7 Å between H5 and 9-CH_3_ clearly distinguishes the correct structure (C) from the C5-epimer (D). The corresponding distributions of distances of the MDOC simulations with effective average distances of 3.8 Å (E) and 4.7 Å (F) are given below.

Conformational flexibility within this molecule may arise from pseudo rotation of the six-membered saturated and lactone rings (rings C and A), as well as the rotation of the methoxy group. MMFF94 conformational analysis showed the boat and chair forms of ring C to be energetically very close (Δ*E* ≈ 0.2 kcal mol^–1^). The vicinal scalar coupling values of *ca.* 7 Hz measured for the protons in the six-membered ring C (see the ESI† for more information) clearly show that averaging between different ring conformations takes place in solution. In addition, some of the cross peaks from the NOESY spectra can only be explained by the presence of a boat-type conformation for the saturated six-membered ring C (see the top of Fig. 5 and 6). Experimental information on ring A is more limited, as only the relatively isolated protons H2 and H5 and the methoxy group are accessible and no defined evidence for the averaging behaviour is available. An NOE contact between the methyl group CH_3_-9 and H5 indicates that the pseudo-equatorial position of the methoxy group should be significantly populated (see the ESI†). Only pseudo-equatorial O–Me conformations were found in the conformational search for the correct *SRRSS* configuration in a large 5.0 kcal mol^–1^ energy window using the MMFF94 force field. Hence the molecular modelling procedure associated with the single tensor SVD approach discards *a priori* any pseudo-axial structures. Note that the universal force field (UFF)^104^ favours a pseudo-axial conformation and DFT M062X supports the pseudo-equatorial conformation, but only with an energy gap Δ*E* = 1.8 kcal mol^–1^. In addition, a close look at the possible conformations of the methyl group in the methoxy moiety reveals that the pseudo-axial conformation implies an almost free rotation about the C5–O axis, while the rotation is severely hindered in the pseudo-equatorial conformer. Therefore a potential entropic contribution favouring the pseudo-axial orientation must be taken into account. In summary, the exact distribution of populations of the different conformers of ring A and the methoxy group are not defined, but it will be likely dominated by a pseudo-equatorial O–Me conformation.

Using the program MSpin, initially a single tensor fitting has been performed for the single lowest energy conformation (see Table 8). In this case, only a poor quality of the fit is achieved (*n*/*χ*^2^ = 0.091) with 10 RDCs being outside the experimental uncertainty, which proves that conformational averaging is present and the corresponding data cannot be represented by a single conformer. Using the full set of four conformers found in the MMFF94 search, the overall fit to experimental RDCs improved dramatically with an overall value of *n*/*χ*^2^ = 1.543 for the best structural ensemble. However, a *χ*–2min value of 0.444 and 3 outliers still violating the experimental data are present, indicating that averaging over additional conformers is necessary. The distribution of the boat *vs.* chair conformation in ring C from the multiple conformer/single tensor fit is *ca.* 1 : 1, which differs from the MMFF94-predicted distribution of approximately 3 : 1. As the selected conformations possess the pseudo-equatorial conformation at the methoxy group, ring A has essentially been treated as rigid.

For MDOC calculations, again, the distances between methylene protons and their respective β-carbons were fixed with the SHAKE algorithm and all C–H vectors were fixed to the COSMOS standard distances for ^1^*D*_CH_ couplings. The pseudo force constant and the scaling factor were optimized for the molecule to *k* = 1.2 10^–3^ kJ Hz^–2^ and *s*_AM_ = 0.002, respectively.

The MDOC simulations for the correct *SRRSS* configuration lead to RDC values which perfectly agree with the experimentally determined values within the experimental uncertainty (see Table 7). A detailed examination of the obtained structural ensembles based on dihedral angles reveals that continuous jumps between different populated conformers occur. Even relatively low pseudo forces can trigger the boat/chair transition, and therefore allow the proper calculation of time averaged RDCs (Fig. 5A and D). A histogram analysis of the dihedral angle H_11β_-C_11_-C_12_-H_12β_ shows the conformational jumps between chair (H_11β_-C_11_-C_12_-H_12β_ < 0) and boat forms (H_11β_-C_11_-C_12_-H_12β_ > 0). It is important to note that jumps take place very frequently within the memory time window *τ*, which ensures that proper time-averaged values are computed. Similarly, monitoring of the MeO-C5-C4-C6 dihedral angle (Fig. 5B and E) shows a distribution of pseudo-equatorial and pseudo-axial positions of the anomeric methoxy groups and frequent jumps between conformers. Monitoring of C4-C5-O-CH_3_ shows that all three angles corresponding to +/– *gauche* and *trans* are populated with a clear preference for +*gauche* and *trans*, reflecting a hindered rotation of the methoxy group in the pseudo-equatorial conformation. As the population of pseudo-axial and pseudo-equatorial conformers is not well-determined, we also performed MDOC simulations with ring A being fixed in the pseudo-equatorial conformation. The resulting structural ensembles equally fitted all experimental constraints within the experimental errors.

**Table 7:** Experimental and computed data for oidiolactone B

Sites	Experiment		MDOC	SVD (single conf.)	SVD (mult. conf)
	*D* ^exp^ ^*a*^/Hz	Δ*D*^*b*^/Hz	〈*D*L*zz*〉_*t*_^*c*^/Hz	*D* ^calc^ ^*d*^/Hz	*D* ^calc^ ^*e*^/Hz
C_2_H_2_	–10.4	0.4	–10.3	–12.0	–10.3
C_5_H_5_	5.8	0.4	5.7	4.1	6.0
C_6_H_6_	–10.1	2.0	–9.9	–11.2	–11.0
C_7_H_7_	–0.8	0.4	–0.8	–0.7	–0.3
C_8_H_8_	18.2	1.0	17.5	11.9	16.7
CH_3_-9	–4.7	1.0	–4.5	–3.1	–4.1
C_10_H_10α_	15.6	1.5	14.3	14.7	16.9
C_10_H_10β_	–1.3	1.0	–1.7	–4.0	–2.3
C_11_H_11α_	0.5*	2.5	0.3	4.9	0.7
C_11_H_11β_	0.8*	2.5	1.8	1.0	1.2
C_12_H_12α_	–0.3	1.0	–0.2	–4.2	–0.8
C_12_H_12β_	10.3	1.5	9.6	11.9	9.1
CH_3_-13	0.4	0.2	0.4	1.7	0.1
CH_3_-5	2.8	0.4	2.8	1.5	2.7

^*a*^Experimental values for ^1^*D*_CH_ residual dipolar couplings.

^*b*^Corresponding experimental maximum error estimates.^93^

^*c*^Time averaged computed RDCs from the MDOC trajectory using the correct assignment.

^*d*^Back calculated RDCs using the single conformer single alignment tensor SVD procedure.

^*e*^Back calculated RDCs using the multiple conformer single alignment tensor SVD procedure.

Altogether sixteen diastereoisomers are possible for oidiolactone B and three methylene groups require diastereotopic assignment. Two of the stereogenic centres are part of the flexible saturated six-membered ring C and one is at the torsionally flexible methoxy group at C5. Conformational spaces were generated for all diastereoisomeric structures at the MMFF94 level. MDOC simulations were started from different conformers, but all simulations lead to basically identical results, indicating that in all cases the experimental constraints were the determining factor for the simulations.

When using the best diastereotopic assignment of CH_2_ groups, the single conformer SVD-based fit for the different configurations results in very low quality factors, indicating in principle the robustness of RDC data interpretation. Also a correct diastereotopic assignment is achieved at methylene groups C10 and C12 – only the almost identical RDCs for the two protons attached to C11 does not allow an unambiguous assignment. The multiple conformer/single tensor fit leads to a further improved distinction of the correct configuration despite the fact that none of the structures fulfils the experimental data within errors. The conformation at C5 in all MMFF94-derived structures is pseudo-equatorial and the corresponding fit to a single alignment tensor helps dramatically in defining all residual stereogenic centres as it fixes the orientation of the tensor axes (see Fig. 6A and B). Due to the restriction to a single alignment tensor and the negligence of vibrational motions, on the other hand, the ability to fully fit experimental data is strongly limited and must result in deviating RDCs. Importantly, the MDOC procedures do not suffer from *a priori* limitations on the selection of particular conformations, associated with the SVD method, although this has the disadvantage of a lower capability for discrimination of the configuration.

In the MDOC simulations the full accessible conformational space is taken into account, leaving no restriction with respect to the number of conformers or the number of alignment tensors being present. In principle, the approach may result in a different alignment tensor for each measured RDC. Again, all sixteen possible configurations of the molecule were compared for their performance using exclusively the fourteen ^1^*D*_CH_ values as MDOC constraints. Based on the *χ*–2min value of the MDOC simulations, the most wrong configurations can be falsified, but a few configurations remain as potential competitors (Table 8). No constraints are violated for the correct stereochemistry (*SRRSS*) and the epimer at C5 (*RRRSS*). A detailed analysis of the structural ensembles reveals that both favour pseudo-equatorial conformations of the methoxy group, in which case the C5-H5 RDC will lead to identical values. However, as no single, common alignment tensor is used in the calculation, the RDC at C5 does not decisively influence the configuration determination in ring C. A change in the configuration at positions C7 and C9 both result in *χ*–2min values slightly below 0.9 with at least one outlier. Apparently, these configurations result in C–H vector orientations that are very similar to the correct structure if the full conformational space is accessible. Nevertheless, a difference larger than 0.4 in *χ*–2min clearly favours the correct configuration.

**Table 8:** Evaluation of the quality criteria for oidiolactone B

Config.^*a*^	MDOC	*χ* –2 min ^*c*^	SVD (single conf.)	SVD (mult. conf.)	SVD (single conf.)	SVD (mult. conf.)
	*n*/*χ*^2^^*b*^		*n*/*χ*^2^^*d*^	*χ* –2 min ^*e*^	*n*/*χ*^2^^*d*^	*χ* –2 min ^*e*^
*SRRSS*	6.788	1.314 (0)	0.091	0.025 (10)	1.543	0.444 (3)
*RRRSS* ^*f*^	6.863	1.367 (0)	0.022	0.002 (9)	0.062	0.017 (10)
*SSRSS*	2.596	0.882 (1)	0.014	0.001 (10)	0.013	0.001 (10)
*SRSSS*	1.304	0.240 (3)	0.013	0.001 (10)	0.012	0.001 (8)
*SRRRS*	4.598	0.866 (1)	0.022	0.007 (13)	0.053	0.005 (7)
*SRRSR* ^*f*^	1.429	0.247 (2)	0.012	0.002 (13)	0.011	0.002 (12)
*RSRSS* ^*f*^	2.526	0.918 (2)	0.009	0.001 (13)	0.014	0.014 (9)
*RRSSS* ^*f*^	1.222	0.230 (3)	0.007	0.001 (11)	0.007	0.001 (11)
*RRRRS* ^*f*^	4.914	0.939 (1)	0.009	0.001 (13)	0.047	0.006 (9)
*RRRSR* ^*f*^	1.272	0.232 (2)	0.007	0.002 (13)	0.005	0.000 (12)
*SSSSS*	1.478	0.220 (4)	0.016	0.004 (13)	0.013	0.002 (12)
*SSRRS*	0.482	0.063 (4)	0.021	0.004 (9)	0.021	0.003 (10)
*SSRSR*	1.021	0.160 (2)	0.016	0.003 (13)	0.015	0.002 (13)
*SRSRS*	0.998	0.117 (4)	0.024	0.006 (8)	0.024	0.003 (12)
*SRSSR*	0.597	0.099 (4)	0.012	0.002 (11)	0.012	0.002 (11)
*SRRRR*	0.999	0.169 (2)	0.017	0.003 (13)	0.016	0.001 (11)
X10	1.544	0.158 (2)	0.017	0.002 (11)	0.018	0.003 (10)
X11	6.943	1.369 (0)	0.094	0.026 (10)	1.446	0.367 (2)
X12	5.087	0.891 (1)	0.030	0.010 (12)	0.118	0.012 (6)

^*a*^Different diastereoisomers given in a five letter code and exchange of prochiral assignment by X and the carbon position.

^*b*^Overall MDOC quality criterion.

^*c*^Strict quality criterion for the MDOC simulation; the number of outliers is given within brackets.

^*d*^Same overall quality criterion computed from the MSpin alignment tensor results.

^*e*^Same strict criterion for MSpin results.

^*f*^Configurations with an inversion of chirality at position C5 can be excluded from the NOE-derived distance between H5 and 9-CH_3_.

With respect to CH_2_ groups, the diastereotopic assignment with MDOC shows the same result as that with MSpin, allowing the correct assignment of methylene protons at C10 and C12, while the very similar RDCs for C11 lead to practically identical results for the two possible assignments. Finally, it should be noted that the *RRRSS* epimer cannot be excluded from one bond RDC data alone, but the structural ensemble from the corresponding MDOC simulations may be used to back calculate other experimentally accessible NMR parameters. Correspondingly, the distance distribution of H5 to the methyl group attached to C9 corresponds well to the measured NOE with an average distance of approximately 3.7 Å for the correct configuration, while the C5 epimer results in a distance distribution of around 4.7 Å (see Fig. 6C–F and annotation f in Table 8).

## 5 Conclusion and outlook

In summary, a novel way of using residual anisotropic NMR parameters as orientational constraints in molecular dynamics simulations (MDOC) is introduced using one-bond residual dipolar couplings (RDCs) as the most easily accessible spectroscopic quantity. In contrast to classical alignment tensor based methods, MDOC is entirely calculated in the laboratory frame, therefore avoiding issues with the treatment of flexibility where a common frame of reference for the alignment tensor is usually not accurately defined. A full description of the underlying theory is given, including rotational averaging, the tensorial force field, and the exponential decay of constraints over time. In the second part of the paper, the possibilities and limitations are explored for several example molecules with different degrees of flexibility.

It should be noted that Salvi *et al.* recently pointed out that methods in the laboratory frame only relying on the projection onto the principal axis of alignment and magnetic field, the *z*-axis, do not allow sufficient angular sampling to produce valid structural ensembles.^105^ The MDOC method, however, is based on tensorial constraints and therefore provides angular sampling with respect to *x*-, *y*-, and *z*-axes as well as the combined *xy*, *yx*, *xz*, *zx*, *yz*, and *zy* components of the dipolar interaction of each coupled spin pair in the laboratory frame. As such, full angular sampling is provided and valid structural ensembles obtained.

**Fig. 7:**
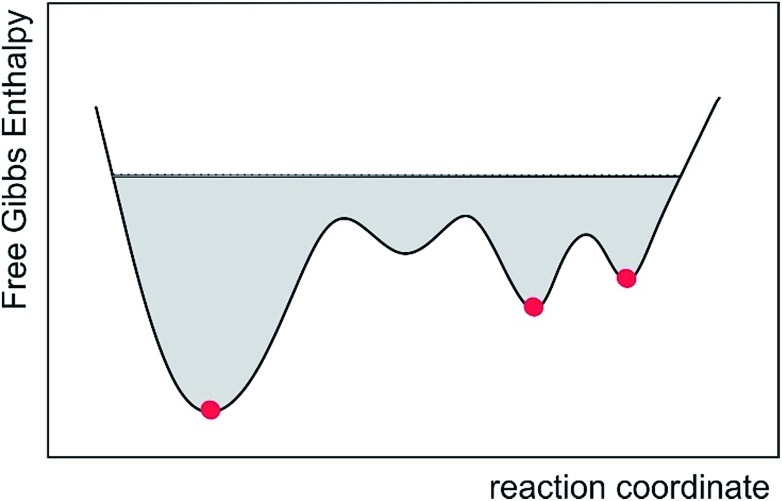
Schematic of the accessible conformational space of the conventional multiple conformer fit and the presented MDOC approach, represented by the commonly used reaction coordinate. While only a few lowest-energy structures are taken into account in the conventional approach (red dots), MDOC takes into account a large area of the potential surface, including entropic contributions (gray area).

During MDOC simulations, rotational averaging of each coupled spin pair is achieved individually *via* pseudo forces mainly originating from non-zero off-diagonal elements of the weighted time averaged dipolar coupling tensor. This directly allows the description of any kind of flexibility as MDOC will rapidly sample the locally available conformational ensemble that best fulfils the NMR parameters. For the correct structural model, the approach usually leads to a structural and orientational ensemble which matches all experimental constraints within the experimental error. If, on the other hand, a structure cannot simultaneously fulfil all experimental constraints, it can be safely neglected. A single “outlier”, *i.e.* a single NMR parameter that does not fulfil experimental data within experimental errors, is sufficient to discriminate or exclude structures. When the approach is applied to the determination of the relative configuration of small molecules, its advantage seems to be the straightforward usage and the avoidance of over interpretation of data as the full available conformational space is sampled. This conformational space *per se* includes structures of low-populated conformations as well as short and long amplitude vibrational contributions. In this way, MDOC simulations also include entropic contributions to the Gibbs free energy (Fig. 7). The MDOC approach, as mentioned above, practically always leads to a very good correspondence of the resulting structural ensemble with experimental constraints, as demonstrated here for a number of cases with known correct structures. On the other hand, the discrimination of configurations is potentially reduced compared to single alignment tensor models since the number of accessible conformers is significantly restricted in the latter. However, this discrimination capability of the SVD approach comes at the expense that even the correct configuration often cannot fulfil experimental constraints within errors, rendering the structural ensemble questionable for further calculations.

Vibrational contributions are not considered and high-energy conformations might be missed out. Even worse, in cases of large structural rearrangements, the single alignment tensor approximation will fail. This will be of particular interest if molecules with flexible chain-like elements,^29^ including *e.g.* intrinsically disordered proteins,^106^ are studied. To avoid such potential misinterpretation, the MDOC approach provides a physically sound and viable alternative as long as sufficient experimental constraints for the molecular dynamics simulations are available. Due to the orientational degeneracy of anisotropic parameters, sparsely conditioned MDOC simulations may lead also to wrong conformations included in a structural ensemble. While this only leads to reduced distinction of relative configurations, with the correct structure still among the allowed ones, the direct interpretation of a structural ensemble as physical reality might be compromised. This effect, however, is not inherent to the method, but depends solely on the number and quality of experimental constraints. As the number of ^1^*D*_CH_ RDCs is usually restricted, we had to limit the application of the MDOC approach to molecules with local flexibility. In all example molecules studied here, the correct configuration could be identified with a structural ensemble that can be used to calculate further molecular properties. For the most flexible molecule under study, oidiolactone B, the fourteen one-bond RDCs alone were not sufficient to exclude the epimer at position C5, but back calculation of the average distance from the resulting structural ensembles and comparison with NOE data clearly excluded the wrong configuration. However, such distances can also be introduced as additional constraints in a molecular dynamics simulation. Even more, isotropic scalar *J*-couplings, by employing a variety of Karplus relationships,^107^ or anisotropic parameters such as long-range RDCs, residual quadrupolar couplings and residual chemical shift anisotropies should be applicable. RCSAs have been measured successfully using a variety of methods,^108^–^110^ and they should result in particularly valuable restraints in protonless spin systems as long as the corresponding CSA tensors can be estimated.^111^,^112^ We are currently working on the corresponding extensions of the COSMOS program. The aim for the future is therefore the inclusion of as many constraints to the MDOC approach as possible. We foresee that most organic molecules, including classes with substantial inherent flexibility, will be amenable to both configurational and conformational analyses using the combined power of experimentally derived anisotropic and isotropic NMR parameters as well as theoretically derived constraints.^113^

## Conflicts of interest

U. S. has written the program COSMOS and was the co-founder of COSMOS-software, Jena, Germany.

## Abbreviations

r-MD: Restrained molecular dynamics
MDOC: Molecular dynamics with orientational constraints
SVD: Singular value decomposition
RDC: Residual dipolar coupling

## Supplementary Material

Supplementary information
