# Supplementary material for: Configuration determination by residual dipolar couplings: accessing the full conformational space by molecular dynamics with tensorial constraints
Source: Chem Sci. 2019 Jul 29;10(38):8774–91. doi: 10.1039/c9sc01084j (PMC6849632; doi:10.1039/c9sc01084j)
Supplement: Supplementary file 1 [file SC-010-C9SC01084J-s001.pdf]

Electronic Supplementary Material (ESI) for Chemical Science.
This journal is © The Royal Society of Chemistry 2019

Supporting Information

Configuration Determination by Residual Dipolar Couplings: Accessing the Full Conformational Space by Molecular Dynamics with Tensorial Constraints

Pavleta Tzvetkova, [‡] Ulrich Sternberg,[#, ‡] Thomas Gloge, Armando Navarro-Vázquez,[†,]* Burkhard Luy*

Institute of Organic Chemistry and Institute for Biological Interfaces 4 – Magnetic Resonance, Karlsruhe Institute of Technology (KIT), Fritz-Haber-Weg 6, 76131 Karlsruhe, Germany

[#] COSMOS-Software, Jena, Germany

[†] Departamento de Química Fundamental, Universidade Federal de Pernambuco, Cidade Universitária, Recife, PE 50740-560, Brazil

[‡] Authors contributed equally

Table of Contents

General information to the MD runs with Orientational Constraints (MDOC) 4

Graphical representation of the tanh (x) 5

Norcamphor 6

Experimental data for norcamphor (1) 6

Coordinates of norcamphor in pdb-file format for the correct configuration 7

Coordinates of norcamphor in coo-file format for the correct configuration 9

Data file for COSMOS MD simulation (.cod) for norcamphor (1) 11

Data file for MSpin SVD fitting calculation for norcamphor 13

MDOC run Project file (.cos) for norcamphor 14

Command to start the MD simulation using the COSMOS backend 20

Synthetic spiroindene derivative 21

Experimental data ($^{1}D_{CH}$) for synthetic spiroindene derivative 2 21

Coordinates files of the spiroindene 22

Coordinates of spiroindene in pdb-file format (wrong configuration) 22

Coordinates of spiroindene in pdb-file format (correct configuration) 23

Coordinates of spiroindene in coo-file format (wrong configuration) 26

Coordinates of spiroindene in coo-file format (correct configuration) 29

Data file for COSMOS MD simulation (.cod) for spiroindene (2) 32

Staurosporine 34

Experimental data for staurosporine 34

Coordinates of staurosporine (3) in pdb file format 35

Coordinates of staurosporine in coo-file format (correct configuration) 38

Data file for MD simulation in program COSMOS for staurosporine 3 41

Oidiolactone B 43

Assignment data of Oidiolactone B 43

Experimental data for Oidiolactone B in PAN/DMSO-d_6 45

Coordinates of Oidiolactone B in pdb file format 46

Coordinates of Oidiolactone B in coo-file format 49

Data file for COSMOS MD simulation (.cod) for oidiolactone B (4) 51

References 53

General information to the MD runs with Orientational Constraints (MDOC)

The molecular dynamics simulations are performed on demonstrative examples of four small molecules with various degrees of flexibility staring from a rigid towards a flexible natural product. The molecules are the following: norcamphor **1**, synthetic spiroindene derivative **2**, a staurosporine **3** and Oidiolactone B **4**.

As the above molecules are used for a principle demonstration set for the capabilities and application of the MDOC their constitution, configuration and the NMR assignment for each molecule was known in advance. For the application the MDOC simulations we have used only one bond C-H residual dipolar couplings (${}^{1}D_{\text{C-H}}$ RDCs). For each MDOC run a coordinate file with the molecular geometry, a data file and a project file are necessary to be prepared for each single molecular model. In the following pages are given the experimental values of the used ${}^{1}D_{\text{C-H}}$ RDCs as orientational constraints, the coordinate file at least for the correct molecular structure, the data file and example project file together with an example command line for starting the MDOC simulation.

Graphical representation of the tanh (x)

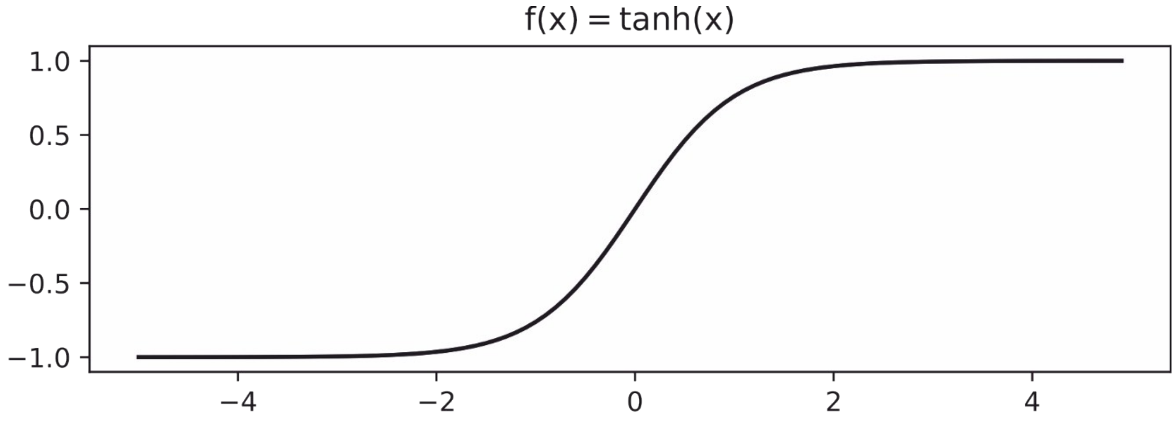

Figure S1. *Graphical representation of the tangent hyperbolic function which represents the potential for the pseudo forces.*

Norcamphor

Experimental data for norcamphor (1)

Table S1. *Experimental data for norcamphor from 0.5 M solution in PEOMMA/TFE-d2 with quadrupolar splitting of 7.6 Hz. Data is obtained from [1]*

	δ_{1H}/[ppm]	δ_{13C}/[ppm]	$^{1}T_{CH}$/[Hz]	$^{1}D_{CH}$/[Hz]	Error [Hz]
C1-H1	2.08	49.4	151.8	1.4	0.5
C3-H3 exo	1.57	44.0	133.5	6.0	0.7
C3-H3 endo	1.33	44.0	139.1	5.0	0.7
C4-H4	2.16	34.5	143.5	-2.9	0.4
C5-H5 exo	1.31	25.6	124.8	-10.5	0.5
C5-H5 endo	0.94	25.6	138.9	5.3	0.5
C6-H6 exo	1.38	22.9	135.9	-1.9	0.6
C6-H6 endo	0.95	22.9	136.0	1.5	0.2
C7-H7 syn	1.27	36.5	139.8	5.8	0.2
C7-H7 anti	1.05	36.5	131.0	-7.1	0.2

Coordinates of norcamphor in pdb-file format for the correct configuration

The coordinates of norcamphor in a pdb-file format generated as an output from the program COSMOS with the correct configuration is given below. This configuration is marked as *Ref* throughout the manuscript.

```
HEADER    NO HEADER DEFINED AND DEFAULT DATE USED        01-JAN-00
AUTHOR    PDB FILE FORMAT GENERATED BY COSMOS
CRYST1    1.000    1.000     1.000   90.000   90.000    90.000 P1
COMPND
REMARK    CREATED FILE CONTAINS ONLY BASIC MOLECULAR DATA
REMARK CELL  1.0000  1.0000  1.0000  90.000  90.000  90.000  0.0000  0.0000  0.0000
REMARK SPGROUP P1
REMARK OPERATION CFF geometry optimization
REMARK ENERGY 273.439
REMARK FORCE_FIELD_ENERGIES BondEn=20.8036 AngleEn=126.713 TorsionEn=128.876
PiTorsionEn=2.40189 CoulombEn=-31.0965 VdWEn=25.7413
REMARK BPT_PSEUDO_ENERGIES IsotropicPsEn=0 TensorialPsEn=0 OrientationalPsEn=0.544335
REMARK NMR_PSEUDO_ENERGIES DistPsEn=0 RDCDistPsEn=0 RDCOrientPsEn=0 JCoupPsEn=0
HETATM    1  O   UNK 0   1       0.641  -2.495  -1.754  1.00  0.00          O
HETATM    2  C4  NOP A   1       0.692   0.211   0.458  1.00  0.00          C
HETATM    3  C6  NOP A   1      -1.027   0.677  -1.146  1.00  0.00          C
HETATM    4  C2  NOP A   1       0.476  -1.429  -1.109  1.00  0.00          C
HETATM    5  C1  NOP A   1      -0.843  -0.822  -0.785  1.00  0.00          C
HETATM    6  C7  NOP A   1      -0.554  -0.678   0.717  1.00  0.00          C
HETATM    7  C5  NOP A   1       0.123   1.357  -0.411  1.00  0.00          C
HETATM    8  C3  NOP A   1       1.588  -0.710  -0.402  1.00  0.00          C
HETATM    9  H1  C1  A   1      -1.702  -1.413  -1.111  1.00  0.00          H
HETATM   10  H3n C3  A   1       2.277  -0.174  -1.059  1.00  0.00          H
HETATM   11  H3x C3  A   1       2.150  -1.380   0.254  1.00  0.00          H
HETATM   12  H4  C4  A   1       1.227   0.599   1.328  1.00  0.00          H
HETATM   13  H5x C5  A   1      -0.258   2.195   0.179  1.00  0.00          H
HETATM   14  H5n C5  A   1       0.887   1.734  -1.095  1.00  0.00          H
HETATM   15  H6n C6  A   1      -1.093   0.949  -2.203  1.00  0.00          H
HETATM   16  H6x C6  A   1      -1.957   0.984  -0.660  1.00  0.00          H
HETATM   17  H7a C7  A   1      -1.346  -0.166   1.269  1.00  0.00          H
HETATM   18  H7s C7  A   1      -0.330  -1.630   1.205  1.00  0.00          H
CONECT    1    4
CONECT    2    8    6    7   12
CONECT    3    7   15    5   16
CONECT    4    1    5    8
CONECT    5    6    3    4    9
CONECT    6   18    5    2   17
CONECT    7    3   13    2   14
CONECT    8   11    4    2   10
CONECT    9    5
CONECT   10    8
CONECT   11    8
CONECT   12    2
CONECT   13    7
CONECT   14    7
```

```
CONECT   15    3
CONECT   16    3
CONECT   17    6
CONECT   18    6
MASTER        0    0    0    0    0    0    0    0

END
```

Coordinates of norcamphor in coo-file format for the correct configuration

The coordinates of norcamphor in a coo-file format generated for the program COSMOS with the correct configuration is given below. This configuration is marked as *Ref* throughout the manuscript.

```
$COO006
CELL  1.0000  1.0000  1.0000  90.000  90.000  90.000  0.0000  0.0000  0.0000
NAME
SPGROUP P1
OPERATION CFF geometry optimization
ENERGY 273.439
FORCE_FIELD_ENERGIES BondEn=20.8036 AngleEn=126.713 TorsionEn=128.876
PiTorsionEn=2.40189 CoulombEn=-31.0965 VdWEn=25.7413
BPT_PSEUDO_ENERGIES IsotropicPsEn=0 TensorialPsEn=0 OrientationalPsEn=0.544335
NMR_PSEUDO_ENERGIES DistPsEn=0 RDCDistPsEn=0 RDCOrientPsEn=0 JCoupPsEn=0
GROUP_TEXT 1 all Carbons
GROUP_TEXT 2 all Protons
GROUP_TEXT 3 DD tensor (constraints)
GROUP_TEXT 4 RDC values
GROUP_TEXT 5 Atoms for fixed distances
GROUP_TEXT 6 13C for RDC calc
GROUP_TEXT 7 tors angles
ATOMS 18
C3 6  1.58809 -0.71014 -0.40197 cmb -0.51130 1 0 8 5 7 16 0 0 0 0
C5 6  0.12278  1.35700 -0.41110 cmb -0.45708 1 0 6 15 7 14 0 0 0 0
C7 6 -0.55366 -0.67813  0.71706 cmb -0.46753 1 0 12 4 7 13 0 0 0 0
C1 6 -0.84256 -0.82227 -0.78499 cmb -0.22383 1 0 3 6 5 11 0 0 0 0
C2 6  0.47600 -1.42925 -1.10866 cmb  0.50124 1 1 18 4 1 0 0 0 0 0
C6 6 -1.02738  0.67737 -1.14644 cmb -0.45246 1 0 2 10 4 9 0 0 0 0
C4 6  0.69181  0.21107  0.45807 cmb -0.17325 1 0 1 3 2 17 0 0 0 0
H3x_C3 1  2.14961 -1.38037  0.25388 cmb  0.23258 1 0 1 0 0 0 0 0 0 0
H6x_C6 1 -1.95710  0.98408 -0.66043 cmb  0.22472 1 0 6 0 0 0 0 0 0 0
H6n_C6 1 -1.09343  0.94930 -2.20301 cmb  0.22348 1 0 6 0 0 0 0 0 0 0
```

```
H1_C1 1 -1.70235 -1.41299 -1.11128 cmb  0.21770 1 0 4 0 0 0 0 0 0 0
H7s_C7 1 -0.33036 -1.63011  1.20545 cmb  0.22514 1 0 3 0 0 0 0 0 0 0
H7a_C7 1 -1.34588 -0.16570  1.26884 cmb  0.22667 1 0 3 0 0 0 0 0 0 0
H5n_C5 1  0.88691  1.73430 -1.09550 cmb  0.22436 1 0 2 0 0 0 0 0 0 0
H5x_C5 1 -0.25807  2.19464  0.17878 cmb  0.22333 1 0 2 0 0 0 0 0 0 0
H3n_C3 1  2.27695 -0.17364 -1.05946 cmb  0.23303 1 0 1 0 0 0 0 0 0 0
H4_C4 1  1.22737  0.59864  1.32848 cmb  0.21129 1 0 7 0 0 0 0 0 0 0
O_UNK_0 8  0.64131 -2.49483 -1.75450 cmb -0.45802 1 1 5 0 0 0 0 0 0 0
```

Data file for COSMOS MD simulation (.cod) for norcamphor (1)

The cosmos data file (*.cod*) containing the experimental data for norcamphor prepared for the MDOC run in the program COSMOS is shown below. The file contains at the beginning lines with remarks (REMARK), which contain information about the conditions and the origin of data, the used groups and their meaning. The experimental RDCs used as constraints follow (DD_TENSORS), showing the used grouping (GROUP_MEMBERS), and fixing of the methylene protons (FIXED_DISTANCES).

<u>Beginning of the COSMOS data file</u>

```
$COD003
REMARK NMR parameters for Norcamphor
REMARK Norcamphor Angew Chem Int Ed Engl 2013, 52, 10309
REMARK RDC from alignment in PEOMMA/TFE-d2
REMARK Group 1: all Carbons
REMARK Group 2: all protons
REMARK Group 3: bond DD tensors - used as constraints (both atoms have to be in
group 1)
REMARK Group 4: RDC values - used as contraints (both atoms have to be in group 4)
REMARK Group 5: fixed distances
REMARK Group 6: 13C for RDC calculation
REMARK Group 7: Torsion angles for Mathematica snapshot
REMARK ordered exp. Values
RC_OPT_MOLECULE IN_MOL
RDC_OPT_MOLECULE IN_MOL
NAMES_OPT_FIT_TO EXACT
DATA_OPT_END
DD_TENSORS 10
H1_C1      C1     -0.7   -0.7    1.4     0      0       0(0.5)
H3x_C3     C3     -3.0   -3.0    6.0     0      0       0(0.7)
H3n_C3     C3     -2.5   -2.5    5.0     0      0       0(0.7)
H4_C4      C4     1.45   1.45   -2.9     0      0       0(0.3)
H5x_C5     C5     5.25   5.25   -10.5    0      0       0(0.5)
H5n_C5     C5     -2.65  -2.65   5.3     0      0       0(0.5)
H6x_C6     C6     0.95   0.95   -1.9     0      0       0(0.5)
H6n_C6     C6     -0.75  -0.75   1.5     0      0       0(0.2)
H7s_C7     C7     -2.9   -2.9    5.8     0      0       0(0.2)
H7a_C7     C7     3.55   3.55   -7.1     0      0       0(0.2)
GROUP_MEMBERS 19
^C.*$                  1 all Carbons
^H.*$                  2 all Protons
H[1-7].*_C[1-7]        3 DD tensor (constraints)
H[1-7].*_C[1-7]        4 RDC values
C[1-7]                 4
H[3].*_C3              5 Atoms for fixed distances
H[5].*_C5              5
H[6].*_C6              5
H[7].*_C7              5
H1_C1                  5
```

```
H4_C4                   5
^C[1-7]                 6 13C for RDC calc
H[34567].*_C[1-7]       7 tors angles
C[1-7]                  7
H[3].*_C3               8 Atoms for fixed distances
H[5].*_C5               8
H[6].*_C6               8
H[7].*_C7               8
C[12456]                8
FIXED_DISTANCES 12
H[3].*_C3 C4
H[3].*_C3 C2
H3x_C3 H3n_C3
H[5].*_C5 C4
H[5].*_C5 C6
H5x_C5 H5n_C5
H[6].*_C6 C5
H[6].*_C6 C1
H6x_C6 H6n_C6
H[7].*_C7 C1
H[7].*_C7 C4
H7s_C7 H7a_C7
END
```

<u>End of COSMOS data file</u>

Data file for MSpin SVD fitting calculation for norcamphor

The data file to be used with the program MSpin to perform the singular value composition (SVD) calculation as a comparison to the outcome from the MD simulation is given below. It contains the same experimental data, but the data file format is different to the COSMOS input data file. The program MSpin allows direct probing of the prochiral assignment using the command permutations where the assignment of the methylene group protons is exchanged automatically during the SVD fit. For easy readability of the data file the atom pair labels are given as a remark, marked with # symbol.

<u>Beginning of the MSpin data file</u>

```
rdc_data {
#H1 C1
5 9 1.400 0.167
#H3x C3
8 11  6.000 0.233
#H3n C3
8 10 5.000 0.233
#H4 C4
2 12 -2.900 0.100
#H5x C5
7 13 -10.500 0.167
#H5n C5
7 14  5.300  0.167
#H6x C6
3 16  -1.900  0.167
#H6n C6
3 15  1.500  0.067
#H7s C7
6 18  5.800  0.067
#H7a C7
6 17 -7.100  0.067
}

permutations {
18 17
}
```

<u>End of MSpin data file</u>

MDOC run Project file (.cos) for norcamphor

The program COSMOS requires a project file, called cosmos options file (*.cos*), which contains all the parameters, which define the MD simulation. The project file allows starting the MD simulations in the background and/or on a cluster environment. The project file is given below on the example of the norcamphor molecule. Some sections of the project file were removed as they are irrelevant to the molecular dynamics simulations used in this manuscript. This ensures better readability of the project file. All the deleted lines are indicated with "…..".

<u>Beginning of the project file</u>

```
//--------------------------------------------------------------------------------
// COSMOS Project File for Version: 6
//--------------------------------------------------------------------------------
// File Names
//--------------------------------------------------------------------------------
PSE_FILE cosmos.pse            //  Periodic System of Elements
FF_PARMFILE forces.par         //  File with non standard force field parameters. If
the entry is empty or forces.par: dafault parameters are used•
Q_PARMFILE q631gnbo.cha        //  Parameters for BPT-charge calculation
BPT_PARMFILE HC_CC-DD.pol      //  Parameters for BPT-property calculation
LOG_FILE COSMOSLog.txt         //  File for Log data output (default: COSMOSLog.txt)
(backend)
OUTPUT_DIR C:\COSMOS_data\NorCamp_best_withFixed_Ref_CH_Ao_HH\ //  Blank|Directory for
output files (front end)
DATA_IMPORT_FILE               //  Blank|Read NMR data and constraints from this *.cod
or *.coo-file (backend)
//--------------------------------------------------------------------------------
// Bond Search Parameters
//--------------------------------------------------------------------------------
...
//--------------------------------------------------------------------------------
// Calculation of Atomic Charges
//--------------------------------------------------------------------------------
ChargeCalcMode 2               //  Mode = 0: No Calculation | 1: EN-Charge | 2: BPT-
Charge
Q_CutoffRadius 30              //  Cutoff radius for the charge calculation
ChargeIncludeSet 0             //  0|n<25 Calculate charges only for this group n
ChargeExcludeSet 0             //  0|n<25 Exclude this group n from charge calculation
ChargedMolecule 0              //  0|1 Search for charged groups in peptides and
proteins
pH_Value 7                     //  pH_Value for charged group search
//--------------------------------------------------------------------------------
// Calculation of Atomic Properties using the Bond Polarization Theory
//--------------------------------------------------------------------------------
BPT_CutoffRadius 30            //  a>0.0: Cutoff radius for BPT property calculations
PCalc_IncludeSet 3             //  0|n<25 calculate properties for this group
```

```
PCalc_ExcludeSet 0             //  0|n<25 exclude this group from property calculation
CS-References                  //  blank|Text with CS references: form El-Mn:name
value (C-13:ppm/TMS 0.0)
BPT_PolIncludeSet 0            //  0|n<25 use only this group for bond polarization
BPT_PolExcludeSet 0            //  0|n<25 exclude this group from bond polarization
TensorCalcMode 1               //  1: Calculate full tensor | 2: Oriented sample
average | 3: Isotropic mean values
SetTensorPoints 3              //  0|n<25 calculate local order tensors for this group
PropertyKeyNumbers 1 3         //  n>0 Numbers of selected data types for the property
calculation
//----------------------------------------------------------------------------
// Parameters for the COSMOS-NMR Force Field
//----------------------------------------------------------------------------
BondLengthOpt 1                //  0|1 Optimize bond length
BondAngleOpt 1                 //  0|1 Optimize bond angles
PiBondTorsionFactor 1          //  a>0 Factor to enhance the pi-torsion barrier
(default 1.0)
DiederAngleOpt 1               //  0|1 Optimize torsion angles
VanDerWaalsOpt 1               //  0|1 Optimize Van der Waals energy
TruncVDW_Repuls 0              //  0|1 Truncate the VdW repulsion to 48 times min
value (version 6)
ElectrostaticOpt 1             //  0|1 Optimize electrostatic energy
BendAngleOpt 1                 //  0|1 Optimize bend angles
AllNBInteractions 1            //  0|1 include all interactions of fixed groups
NB_ElectrostaticsOnly 0        //  0|1 include only electrostatics of fixed groups
(version 6)
ELS_ForceMemoryTimeC 100       //  NB_ElectrostaticsOnly?: Memory time (ps) for
electrostatic forces
FF_IncludeSet 0                //  0|n<25 run force field for this connected group
only
FF_ExcludeSet 0                //  0|n<25 exclude this connected group from force
field
FF_FixSet 0                    //  0|n<25 fix this group of atoms
FF_FixedBondsSet 5             //  0|n<25 fix all bond lengths for this group in MD
simulations
MD_FixedDistancesSet 8         //  0|n<25 fix distances as given in R-constraints for
this group in MD simulations(version 6)
FF_UnitCellSet 0               //  0|n<25 group of unit cell content
CutoffRadVDW 6                 //  Cutoff radius for Van der Waals interactions
CutoffRadELS 30                //  Cutoff radius for electrostatic interactions
HarmonicBondPotential 1        //  0|1: Use harmonic potentials for bond forces
PeriodicBoundaryCond 0         //  0|1: Periodic box
CrystBoundaryCond 0            //  0|1: Periodic crystal lattice
BPT-PeriodicCell/Box 0         //  0|1: BPT calculation for periodic box or cell
ShiftMolToCrystCell 0          //  0|1: Shift molecules to cell in MD calculations
//----------------------------------------------------------------------------
// Geometry Optimization using the COSMOS-NMR Force Field
//----------------------------------------------------------------------------
MaxCycles 10000                //  n: Maximum number of cycles
PictureCycles 1                //  0|n: Refresh picture after n cycles (front end)
ControlCycles 10               //  0|n: Generate log output after n cycles (front end)
ChargeCycles 1                 //  0|n: Calculate BPT charges after n cycles
OptimizationMode 1             //  0: Gradient optimization | 1: conjugated gradient
optimization
StartSlope 1                   //  a>0 Optimization step width at start (default 1.0)
```

```
ForceEps 1e-06                 //  fabs(forces(cycle n)-forces(cycle n-1))<eps ?: terminate optimization
//-------------------------------------------------------------------------------
// Snapshots in Molecular Dynamics Simulations using the COSMOS-NMR Force Field
//-------------------------------------------------------------------------------
SnapshotTypes COO DIST TORSION DD_TENS ENERGY //  TYPE TYPE ...TYPE=COO|DIST|ANGLES|TORSION|RAMA|INERTIA|CHARGES|CS_ISO|CS_TENS|DD_TENS|QC_TENS|RDC|JC|ENERGY
COO_SnapGroup 0                //  0|n<25 Group for coordinate (COO) snapshot
DIST_SnapGroupFrom 1           //  0|n<25 Distance (DIST) snapshots from this group n of atoms
DIST_SnapGroupTo 1             //  0|n<25 Distance (DIST) snapshots to this group n of atoms
DIST_SnapCutoff 4              //  a>0.0 Cutoff radius a for distance (DIST) snapshots
ANGLES_SnapGroup 0             //  0|n<25 Angles (ANGLES) snapshots from this group n of atoms
TORSIONS_SnapGroup 7           //  0|n<25 Torsion angles (TORSION) snapshots from this group n of atoms
RAMA_SnapGroup 0               //  0|n<25 Ramachandran (RAMA) snapshots from this group n of atoms
CHARGES_SnapGroup 0            //  0|n<25 Atomic charges (CHARGES) snapshots from this group n of atoms
CS_SnapGroup 0                 //  0|n<25 Chemical shift (CS_ISO) snapshots from this group n of atoms
BPT_TensorGroup 0 3 0          //  0|n<25 BPT tensor (CS, DD, QC) snapshots from this group n of atoms
RDC_SnapGroup 4                //  0|n<25 RDC-Coupling snapshots from this group n of atoms, group 0:all calculated RDC
J_SnapGroup 0                  //  0|n<25 J-Coupling snapshots from this group n of atoms
//-------------------------------------------------------------------------------
// Property Calculation Controls in MD Simulations using the COSMOS-NMR Force Field
//-------------------------------------------------------------------------------
GroupFixedBondLength 5         //  0|n<25 Fix the bond length for this group n (set parameter FF_FixedBondsSet for force field)
PropertyCycles 1               //  0|n: Calculate NMR properties after n cycles
EnablePropertyCalc 1           //  0|1: Enable BPT property calculation
Enable_NMR_Spec 0              //  0|1: Enable NMR spectra display (front end)
NMR_SpecCycles 10              //  n Cycles for NMR spectra refresh (front end)
TimeAverageProperty 1          //  0|1: Calculate time average of BPT properties
PropertyMemoryTimeConst 200    //  a>0.0 in pico sec: memory time constant for the property average
//-------------------------------------------------------------------------------
// Temperature Controls in MD Simulations using the COSMOS-NMR Force Field
//-------------------------------------------------------------------------------
ThermostatingMode 1            //  0:No thermostating | 1:Keep temperature constant | 2: Heating or cooling
RestartMode 1                  //  0:Restart at actual temperature | 1:Start with random velocities
StartTemperature 300           //  a>0.0 Start temperature in Kelvin in the case of RestartMode=1
TargetTemperature 300          //  a>0.0 Target temperature in Kelvin (heating or cooling)
T_CouplingTimeConst 0.02       //  a>0.0 Coupling time constant to the thermostat in pico sec
```

```
//--------------------------------------------------------------------------------
// Process Controls in MD Simulations using the COSMOS-NMR Force Field
//--------------------------------------------------------------------------------
DynaPicCycles 100              //  0|n Cycles for the picture refresh (front end)
DynaControlCycles 200          //  0|n Cycles for Log-window output or programmed stop
DataSnapshotCycles 10000       //  0|n Cycles for data snapshots
MaxTimeSteps 20000000          //  n>0 Maximum number of time steps
FemtoTimeStep 0.5              //  a>0.0 Verlet time step in femto sec.
DynChargeCycles 4              //  0|n Cycles for BPT atomic charge calculation
CoordSnapshotCycles 20000      //  0|n Cycles for coordinate snapshots
HeatingCoolingCycles 298       //  0<n<=MaxTimeSteps: Cycles for heating or cooling
(only if ThermostatingMode==2)
//--------------------------------------------------------------------------------
// MPI Controls in MD Simulations using the COSMOS-NMR Force Field
//--------------------------------------------------------------------------------
GlobalRemoveTransRotCyc 1000   //  n Cycles for global remove of translations or
rotation (MPI backend only)
GlobalThermostatingCyc 1000    //  n Cycles for global thermostating (MPI backend
only)
GlobalExchangeChargesCyc 100   //  n Cycles for global BPT charge exchange (MPI
backend only)
ProcessPartitionMethod 0       //  0:Process partition of molecules |1:Sequential
partition
//--------------------------------------------------------------------------------
// Spectra and Distributions Controls in MD Simulations using the COSMOS-NMR FF
//--------------------------------------------------------------------------------
SaveNMR_Spectra 0              //  n Cycles to save NMR spectra (front end)
SaveDistributionFkt 0          //  n Cycles to save distribution functions (front end)
EnableCalcDistFkt 0            //  0|1: Enable the calculation of distribution
functions (front end)
EnableShowDistFkt 0            //  0|1: Enable the display of distribution functions
(front end)
EnableResetMotions 1           //  0|1: Enable the elimination of global motions
PutAllMoleculesToBox 0         //  1: All molecules are put to the periodic box |0:
Mol. with atoms outside the box are not treated periodically
//--------------------------------------------------------------------------------
// Table Output
//--------------------------------------------------------------------------------
AtNbInCoordTable 1             //  0|1: Numbering of atoms in table
SymbInCoordTable 1             //  0|1: Site name in table
PseNbInCoordTable 1            //  0|1: PSE number in table
CoordTypeInTable 0             //  0: Cartesian | 1: crystallographic coordinates
(front end)
AtNbInChargeTable 1            //  0|1: Numbering in charge table
SymbInChargeTable 1            //  0|1: Site name in charge table
PseNbInChargeTable 1           //  0|1: PSE number in charge table
CSIsoTable 1                   //  0|1: Display isotropic property table
CSTensorTable 0                //  0|1: Display tensorial property table
CSAnisoTable 1                 //  0|1: Display anisotropy parameters
SelectedTableType 0            //  0: All atoms | 1: Group | 2: Surrounding of atom in
property table
CentralAtInTable 1             //  0|n: Central atom n for surrounding for table type
2
GroupInTable 0                 //  0|n: Group n for table type 1
RadiusInTable 1e+03            //  r>0 Radius for atom surrounding for table type 2
```

```
GeoOptEnergyLog 1              //  0|1: Output of running geometry optimization to
log-window (front end) or log-file
DynamicsOutputLog 1            //  0|1: Output of running MD info to log-window (front
end) or log-file
MonteCarloOutputLog 0          //  0|1: Output of Monte-Carlo info to log-window
(front end) or log-file
TableOutputType CONSTR RCON JCON RDC //  TYPE TYPE...TYPE=CHAR|BPT-PROP|JC|TENS-
O|PISEMA|ORDER|DIP|CONSTR|RDC|RCON|JCON|PSEUDO-EN|ENERGY
//--------------------------------------------------------------------------------
// Fit of Molecular Structures to Properties and NMR-Spectra
//--------------------------------------------------------------------------------
.....
//--------------------------------------------------------------------------------
// Calculation of BPT Pseudo-Forces for NMR Structure Fit
//--------------------------------------------------------------------------------
....
//--------------------------------------------------------------------------------
// Width and Weight Parameters for Distance Constraints
//--------------------------------------------------------------------------------
....
//--------------------------------------------------------------------------------
// Width and Weight Parameters for Orientational Pseudo-Forces
//--------------------------------------------------------------------------------
CS_TensorWidth 0               //  a>0 Width parameter for the oriented CS constraints
fit
CS_TensorWeight 0              //  a>0 Weight factor for the oriented CS pseudo forces
DD_TensorWidth 0.5             //  a>0 Width parameter for the oriented DD constraints
fit
DD_TensorWeight 0.0006         //  a>0 Weight factor for the oriented DD pseudo forces
Q_TensorWidth 0                //  a>0 Width parameter for the oriented QC constraints
fit
Q_TensorWeight 0               //  a>0 Weight factor for the oriented QC pseudo forces
//--------------------------------------------------------------------------------
// Calculation of Distribution Functions
//--------------------------------------------------------------------------------
DistanceDistrDataPoints 600    //  n: Number of data points in distance distribution
graphics
CalcDistDitribution 0          //  0:1 Calculate distance distribution (front end V6)
DistanceDistrSearchRad 6       //  a>0 Search radius for distance distribution
DistanceGroup 0                //  0|n<25 Group for distance distribution
DistanceGroupTo 0              //  0|n<25 Second group for distance distribution
CalcBondAnglesDist 0           //  0:1 Calculate bond angle distribution graphics
(front end V6)
BondAngleGroup 0               //  0|n<25 Group for bond angle distribution
BondAngleMin 0                 //  a: Minimum in bond angle graphics
BondAngleMax 180               //  a: Maximum in bond angle graphics
CalcTorsionAnglesDist 0        //  0:1 Calculate torsion angle distribution graphics
(front end V6)
TorsionAngleGroup 0            //  0|n<25 Group for torsion angle distribution
TorsionAngleMin -180           //  a: Minimum in torsion angle graphics
TorsionAngleMax 180            //  a: Maximum in torsion angle graphics
BondAngleDistType 0            //  0: General angle distribution | 1: Bond angle
distribution
//--------------------------------------------------------------------------------
// Display of Chemical Shift Spectra
```

```
//---------------------------------------------------------------------------
....
//---------------------------------------------------------------------------
// Display of Quadrupolar Spectra
//---------------------------------------------------------------------------
....
//---------------------------------------------------------------------------
// Display of Dipolar Spectra
//---------------------------------------------------------------------------
.....
//---------------------------------------------------------------------------
// Width and Weight Factors of the RDC Distance Fit
//---------------------------------------------------------------------------
.....
//---------------------------------------------------------------------------
// Width and Weight Factors of the Orientational RDC Fit
//---------------------------------------------------------------------------
.....
//---------------------------------------------------------------------------
// RDC Calculation
//---------------------------------------------------------------------------
.....
//---------------------------------------------------------------------------
// Oriented Sample
//---------------------------------------------------------------------------
OrientedTensCalcMode 1         //  0:None |1: Full tensor |2: Mean value for fast
rotation around director |3: Full tensor + rot. distribution
DirectorTiltAngle 0            //  0<= a <=180 Director tilt angle
SampleOrderParameter 0.001     //  0<= a <=1.0 Order parameter of oriented sample
//---------------------------------------------------------------------------
// Global Width Settings
//---------------------------------------------------------------------------
UseErrorsAsWidth 1             //  0|1 Use the experimental errors as width for DD,
RDC, R and J constraints in MD simulations
//---------------------------------------------------------------------------
// Import and Export Options
//---------------------------------------------------------------------------
.....
//---------------------------------------------------------------------------
// Parameters for 3J Couplings
//---------------------------------------------------------------------------
.....
```

<u>End project file</u>

Command to start the MD simulation using the COSMOS backend

The command line for starting the MDOC run in the background and/or in a cluster environment is given below on the example of norcamphor. It indicates the location of all the necessary files: program executable, project file (.*cos*), data file (.*cod*), configuration directory, data directory to store the output files and the cosmos coordinate file (.*coo*).

Beginning of the script

```
/home/kit/USER/COSMOS/bin/cosback.x86_64-STATIC -dw -c
/home/kit/USER/Norcamphor/NorCamp_best_withFixed_Ref_CH_Ao_HH_0.00045DD_width.cos
-a
/home/kit/USER/Norcamphor/norcamphor_withFixed_Ao_HH_cor.cod -f
/home/kit/USER/COSMOS/config -y
/work/kit/USER/NorCamp_best_withFixed_Ref_CH_Ao_HH_0.00045DD_width_corr_best_Ref_
CH_Ao
/home/kit/USER/Norcamphor/norcamphor_raw_opt_best_Ref_CH_Ao.coo
```

End of the script

Synthetic spiroindene derivative

Experimental data ($^1D_{CH}$) for synthetic spiroindene derivative 2

Table S2. *Experimental one bond $^1D_{CH}$ in Hz for synthetic spiroindene derivative in PDMS/CDCl3 as given in [2], where the experimental errors in Hz are obtained directly from the authors.*

	δ_{1H}/[ppm]*	δ_{13C}/[ppm]	$^1T_{CH}$/[Hz]	$^1D_{CH}$/[Hz]	Error [Hz]
C3-H3				-0.30	1.0
C4-H4				4.90	1.0
C5-H5				-2.05	2.0
C2-H2				-2.20	2.0
C1a-H1a				-2.00	2.0
C6-Ha6				-1.90	1.0
C6-Hb6				-3.50	1.0
C6a-H6a				-0.20	2.0
C4’-H4’				-1.70	1.0
C5’-H5’				6.40	1.0
C6’-H6’				-1.25	1.5
C7’-H7’				-1.10	1.0

*- the chemical shift data as well as the total one-bond coupling is not available from the used literature sources.

Coordinates files of the spiroindene

As in the case of the spiroindene molecule **2**, only two possible relative configurations are possible.

Corresponding pdb-files for the right and the wrong configuration are given below:

Coordinates of spiroindene in pdb-file format (wrong configuration)

```
HEADER    NO HEADER DEFINED AND DEFAULT DATE USED        01-JAN-00
AUTHOR    PDB FILE FORMAT GENERATED BY COSMOS
CRYST1    1.000    1.000     1.000   90.000   90.000    90.000 P1
COMPND
REMARK    CREATED FILE CONTAINS ONLY BASIC MOLECULAR DATA
REMARK CELL  1.0000  1.0000  1.0000  90.000  90.000  90.000  0.0000  0.0000
0.0000
REMARK SPGROUP P1
HETATM    1  C2P NOP A   1       0.808   2.039   1.225  1.00  0.00           C
HETATM    2  C6P NOP A   1       0.943  -2.652   2.569  1.00  0.00           C
HETATM    3  C5P NOP A   1       2.329  -2.352   2.653  1.00  0.00           C
HETATM    4  C3  NOP A   1      -3.562  -2.208  -0.666  1.00  0.00           C
HETATM    5  C4  NOP A   1      -4.247  -2.448   0.556  1.00  0.00           C
HETATM    6  C4P NOP A   1       2.797  -1.048   2.349  1.00  0.00           C
HETATM    7  C3P NOP A   1       2.183   1.401   1.587  1.00  0.00           C
HETATM    8  C6  NOP A   1      -2.650   0.420   2.674  1.00  0.00           C
HETATM    9  C5  NOP A   1      -4.013  -1.624   1.688  1.00  0.00           C
HETATM   10  C2  NOP A   1      -2.633  -1.140  -0.775  1.00  0.00           C
HETATM   11  C5a NOP A   1      -3.089  -0.560   1.571  1.00  0.00           C
HETATM   12  C1b NOP A   1      -2.406  -0.323   0.358  1.00  0.00           C
HETATM   13  C6a NOP A   1      -1.661   1.370   1.963  1.00  0.00           C
HETATM   14  C1a NOP A   1      -1.459   0.880   0.443  1.00  0.00           C
HETATM   15  C7P NOP A   1       0.007  -1.656   2.184  1.00  0.00           C
HETATM   16  C3a NOP A   1       1.857  -0.061   1.968  1.00  0.00           C
HETATM   17  C1P NOP A   1      -0.243   0.927   1.443  1.00  0.00           C
HETATM   18  C7a NOP A   1       0.474  -0.352   1.890  1.00  0.00           C
HETATM   19  H1a C1a A   1      -1.606   1.712  -0.250  1.00  0.00           H
HETATM   20  H2  C2  A   1      -2.103  -0.950  -1.712  1.00  0.00           H
HETATM   21  HA2PC2P A   1       0.803   2.371   0.184  1.00  0.00           H
HETATM   22  HB2PC2P A   1       0.602   2.895   1.873  1.00  0.00           H
HETATM   23  H3  C3  A   1      -3.750  -2.852  -1.529  1.00  0.00           H
HETATM   24  HB3PC3P A   1       2.866   1.419   0.734  1.00  0.00           H
HETATM   25  HA3PC3P A   1       2.646   1.910   2.437  1.00  0.00           H
HETATM   26  H4  C4  A   1      -4.956  -3.277   0.626  1.00  0.00           H
HETATM   27  H4P C4P A   1       3.862  -0.807   2.404  1.00  0.00           H
HETATM   28  H5  C5  A   1      -4.528  -1.810   2.635  1.00  0.00           H
HETATM   29  H5P C5P A   1       3.038  -3.129   2.949  1.00  0.00           H
HETATM   30  HB6 C6  A   1      -3.515   0.972   3.051  1.00  0.00           H
HETATM   31  HA6 C6  A   1      -2.159  -0.105   3.498  1.00  0.00           H
HETATM   32  H6P C6P A   1       0.594  -3.661   2.801  1.00  0.00           H
HETATM   33  H6a C6a A   1      -1.849   2.445   2.025  1.00  0.00           H
HETATM   34  H7P C7P A   1      -1.053  -1.916   2.122  1.00  0.00           H
CONECT    1   22   17    7   21
CONECT    2   15    3   32
```

```
CONECT    3    6    2   29
CONECT    4   10    5   23
CONECT    5    9    4   26
CONECT    6   16    3   27
CONECT    7   16   24    1   25
CONECT    8   13   30   11   31
CONECT    9   11    5   28
CONECT   10   12    4   20
CONECT   11    8    9   12
CONECT   12   10   11   14
CONECT   13   17    8   14   33
CONECT   14   17   12   13   19
CONECT   15   18    2   34
CONECT   16    6    7   18
CONECT   17    1   13   14   18
CONECT   18   15   16   17
CONECT   19   14
CONECT   20   10
CONECT   21    1
CONECT   22    1
CONECT   23    4
CONECT   24    7
CONECT   25    7
CONECT   26    5
CONECT   27    6
CONECT   28    9
CONECT   29    3
CONECT   30    8
CONECT   31    8
CONECT   32    2
CONECT   33   13
CONECT   34   15
MASTER        0    0    0    0    0    0    0    0
END
```

Coordinates of spiroindene in pdb-file format (correct configuration)

```
HEADER    NO HEADER DEFINED AND DEFAULT DATE USED        01-JAN-00
AUTHOR    PDB FILE FORMAT GENERATED BY COSMOS
CRYST1    1.000    1.000     1.000   90.000   90.000    90.000 (null)
COMPND
REMARK    CREATED FILE CONTAINS ONLY BASIC MOLECULAR DATA
REMARK CELL  1.0000  1.0000  1.0000  90.000  90.000  90.000  0.0000  0.0000
0.0000
HETATM    1  C5P NOP A   1       3.423  -0.988  -2.122  1.00  0.00           C
HETATM    2  C6P NOP A   1       2.377  -0.277  -2.771  1.00  0.00           C
HETATM    3  C3  NOP A   1      -4.290  -1.486   2.198  1.00  0.00           C
HETATM    4  C4  NOP A   1      -4.436  -0.344   3.031  1.00  0.00           C
HETATM    5  C4P NOP A   1       3.335  -1.301  -0.739  1.00  0.00           C
HETATM    6  C3P NOP A   1       1.879  -1.128   1.474  1.00  0.00           C
HETATM    7  C6  NOP A   1      -1.694   1.912   1.396  1.00  0.00           C
HETATM    8  C5  NOP A   1      -3.626   0.806   2.831  1.00  0.00           C
HETATM    9  C7P NOP A   1       1.223   0.132  -2.052  1.00  0.00           C
HETATM   10  C2  NOP A   1      -3.332  -1.496   1.150  1.00  0.00           C
```

```
HETATM   11  C3a NOP A   1       2.184  -0.893  -0.024  1.00  0.00           C
HETATM   12  C5a NOP A   1      -2.675   0.789   1.784  1.00  0.00           C
HETATM   13  C1b NOP A   1      -2.532  -0.345   0.954  1.00  0.00           C
HETATM   14  C6a NOP A   1      -0.904   1.336   0.198  1.00  0.00           C
HETATM   15  C1a NOP A   1      -1.435  -0.153  -0.099  1.00  0.00           C
HETATM   16  C7a NOP A   1       1.146  -0.190  -0.676  1.00  0.00           C
HETATM   17  C2P NOP A   1       0.508  -0.425   1.731  1.00  0.00           C
HETATM   18  C1P NOP A   1       0.052   0.096   0.352  1.00  0.00           C
HETATM   19  H1a C1a A   1      -1.668  -0.287  -1.159  1.00  0.00           H
HETATM   20  H2  C2  A   1      -3.210  -2.372   0.509  1.00  0.00           H
HETATM   21  HB2PC2P A   1       0.633   0.408   2.428  1.00  0.00           H
HETATM   22  HA2PC2P A   1      -0.214  -1.134   2.144  1.00  0.00           H
HETATM   23  H3  C3  A   1      -4.918  -2.364   2.366  1.00  0.00           H
HETATM   24  HA3PC3P A   1       1.816  -2.201   1.671  1.00  0.00           H
HETATM   25  HB3PC3P A   1       2.671  -0.690   2.087  1.00  0.00           H
HETATM   26  H4  C4  A   1      -5.177  -0.350   3.835  1.00  0.00           H
HETATM   27  H4P C4P A   1       4.137  -1.848  -0.234  1.00  0.00           H
HETATM   28  H5  C5  A   1      -3.731   1.686   3.471  1.00  0.00           H
HETATM   29  H5P C5P A   1       4.303  -1.297  -2.691  1.00  0.00           H
HETATM   30  HB6 C6  A   1      -2.249   2.805   1.099  1.00  0.00           H
HETATM   31  HA6 C6  A   1      -1.026   2.152   2.228  1.00  0.00           H
HETATM   32  H6P C6P A   1       2.462  -0.043  -3.835  1.00  0.00           H
HETATM   33  H6a C6a A   1      -0.801   1.939  -0.708  1.00  0.00           H
HETATM   34  H7P C7P A   1       0.417   0.677  -2.550  1.00  0.00           H
CONECT    1    5    2   29
CONECT    2    9    1   32
CONECT    3   10    4   23
CONECT    4    8    3   26
CONECT    5   11    1   27
CONECT    6   25   17   11   24
CONECT    7   31   14   12   30
CONECT    8   12    4   28
CONECT    9   16    2   34
CONECT   10   13    3   20
CONECT   11    5    6   16
CONECT   12    7    8   13
CONECT   13   10   12   15
CONECT   14   18    7   15   33
CONECT   15   18   13   14   19
CONECT   16    9   11   18
CONECT   17   22   18    6   21
CONECT   18   14   15   16   17
CONECT   19   15
CONECT   20   10
CONECT   21   17
CONECT   22   17
CONECT   23    3
CONECT   24    6
CONECT   25    6
CONECT   26    4
CONECT   27    5
CONECT   28    8
CONECT   29    1
CONECT   30    7
CONECT   31    7
```

```
CONECT   32    2
CONECT   33   14
CONECT   34    9
MASTER        0    0    0    0    0    0    0    0
END
```

Coordinates of spiroindene in coo-file format (wrong configuration)

The coordinates of spiroindene in a coo-file format generated as an input for the program COSMOS with the wrong configuration is given below.

```
$COO006
CELL  1.0000  1.0000  1.0000  90.000  90.000  90.000  0.0000  0.0000  0.0000
NAME
SPGROUP P1
GROUP_TEXT 1 all Carbons
GROUP_TEXT 2 all Protons
GROUP_TEXT 3 DDs
GROUP_TEXT 5 Fix CH-bond
GROUP_TEXT 6 13C for RDC calculation
GROUP_TEXT 7 Torsion angles
GROUP_TEXT 8 Fix distances
ATOMS 34
C7aP 6  0.47428 -0.35190  1.88971 smb 193 1 3 4 3 2 0 0 0 0 0
C1P 6 -0.24254  0.92720  1.44303 smb 193 1 0 18 6 5 1 0 0 0 0
C3aP 6  1.85651 -0.06094  1.96756 smb 193 1 5 13 12 1 0 0 0 0 0
C7P 6  0.00684 -1.65568  2.18437 smb 33 1 3 1 17 34 0 0 0 0 0
C1a 6 -1.45907  0.88012  0.44308 smb 225 1 0 2 7 6 19 0 0 0 0
C6a 6 -1.66130  1.37046  1.96251 smb 225 1 0 2 11 5 20 0 0 0 0
C1b 6 -2.40570 -0.32260  0.35764 smb 65 1 3 9 8 5 0 0 0 0 0
C5a 6 -3.08894 -0.56024  1.57067 smb 193 1 6 11 10 7 0 0 0 0 0
C2 6 -2.63303 -1.13989 -0.77499 smb 33 1 3 7 15 33 0 0 0 0 0
C5 6 -4.01311 -1.62437  1.68839 smb 33 1 3 8 14 32 0 0 0 0 0
C6 6 -2.65005  0.42000  2.67438 smb 97 1 0 6 22 8 21 0 0 0 0
C3P 6  2.18327  1.40132  1.58723 smb 193 1 0 3 31 18 30 0 0 0 0
C4P 6  2.79706 -1.04795  2.34913 smb 33 1 3 3 16 29 0 0 0 0 0
C4 6 -4.24716 -2.44819  0.55557 smb 33 1 3 10 15 28 0 0 0 0 0
C3 6 -3.56164 -2.20805 -0.66556 smb 33 1 3 9 14 27 0 0 0 0 0
```

```
C5P 6  2.32890 -2.35189  2.65293 smb 33 1 3 13 17 26 0 0 0 0 0

C6P 6  0.94284 -2.65156  2.56871 smb 33 1 3 4 16 25 0 0 0 0 0

C2P 6  0.80812  2.03927  1.22504 smb 193 1 0 23 2 12 24 0 0 0 0

H1a_C1a 1 -1.60574  1.71206 -0.25047 smb 22 1 0 5 0 0 0 0 0 0 0

H6a_C6a 1 -1.84860  2.44546  2.02536 smb 22 1 0 6 0 0 0 0 0 0 0

Ha6_C6 1 -2.15924 -0.10519  3.49774 smb 150 1 0 11 0 0 0 0 0 0 0

Hb6_C6 1 -3.51498  0.97191  3.05114 smb 150 1 0 11 0 0 0 0 0 0 0

Hb2P_C2P 1  0.60180  2.89529  1.87259 smb 146 1 0 18 0 0 0 0 0 0 0

Ha2P_C2P 1  0.80350  2.37114  0.18366 smb 146 1 0 18 0 0 0 0 0 0 0

H6P_C6P 1  0.59384 -3.66099  2.80090 smb 22 1 0 17 0 0 0 0 0 0 0

H5P_C5P 1  3.03797 -3.12926  2.94884 smb 22 1 0 16 0 0 0 0 0 0 0

H3_C3 1 -3.74978 -2.85156 -1.52878 smb 22 1 0 15 0 0 0 0 0 0 0

H4_C4 1 -4.95575 -3.27738  0.62618 smb 22 1 0 14 0 0 0 0 0 0 0

H4P_C4P 1  3.86182 -0.80722  2.40357 smb 22 1 0 13 0 0 0 0 0 0 0

Ha3P_C3P 1  2.64624  1.90982  2.43679 smb 146 1 0 12 0 0 0 0 0 0 0

Hb3P_C3P 1  2.86581  1.41915  0.73372 smb 146 1 0 12 0 0 0 0 0 0 0

H5_C5 1 -4.52765 -1.80990  2.63469 smb 22 1 0 10 0 0 0 0 0 0 0

H2_C2 1 -2.10349 -0.94969 -1.71204 smb 22 1 0 9 0 0 0 0 0 0 0

H7P_C7P 1 -1.05276 -1.91634  2.12154 smb 22 1 0 4 0 0 0 0 0 0 0

SITE_NAMES

 1 C7aP                   18 C2P                   19 H1a_C1a                20
H6a_C6a                21 Ha6_C6

 22 Hb6_C6                 23 Hb2P_C2P              24 Ha2P_C2P               25
H6P_C6P                26 H5P_C5P

 27 H3_C3                  28 H4_C4                 29 H4P_C4P                30
Ha3P_C3P               31 Hb3P_C3P

 32 H5_C5                  33 H2_C2                 34 H7P_C7P

DD_TENSORS 12

19 5    1.00000    1.00000   -2.00000    0.00000    0.00000    0.00000(2)

20 6    0.10000    0.10000   -0.20000    0.00000    0.00000    0.00000(2)

21 11    0.95000    0.95000   -1.90000    0.00000    0.00000    0.00000(1)

22 11    1.75000    1.75000   -3.50000    0.00000    0.00000    0.00000(1)

25 17    0.62500    0.62500   -1.25000    0.00000    0.00000    0.00000(1.5)

26 16   -3.20000   -3.20000    6.40000    0.00000    0.00000    0.00000(1)

27 15    0.15000    0.15000   -0.30000    0.00000    0.00000    0.00000(1)
```

```
28 14   -2.45000   -2.45000    4.90000    0.00000    0.00000    0.00000(1)
29 13    0.85000    0.85000   -1.70000    0.00000    0.00000    0.00000(1)
32 10    1.02500    1.02500   -2.05000    0.00000    0.00000    0.00000(2)
33 9    1.10000    1.10000   -2.20000    0.00000    0.00000    0.00000(2)
34 4    0.55000    0.55000   -1.10000    0.00000    0.00000    0.00000(1)
FIXED_DISTANCES 8
1 5 2.7108 6 2.74454
18 5 2.66369 6 2.66256
21 6 2.18687 8 2.18747 22 1.78819
22 6 2.18634 8 2.17274
23 2 2.18422 12 2.19418
24 2 2.18293 12 2.19416 23 1.77986
30 3 2.17434 18 2.2054 31 1.78589
31 3 2.17525 18 2.20455
```

Coordinates of spiroindene in coo-file format (correct configuration)

The coordinates of spiroindene in a coo-file format generated as an input for the program COSMOS with the correct configuration is given below.

```
$COO006
CELL  1.0000  1.0000  1.0000  90.000  90.000  90.000  0.0000  0.0000  0.0000
NAME
GROUP_TEXT 1 all Carbons
GROUP_TEXT 2 all Protons
GROUP_TEXT 3 DDs
GROUP_TEXT 5 Fix CH-bond
GROUP_TEXT 6 13C for RDC calculation
GROUP_TEXT 7 Torsion angles
GROUP_TEXT 8 Fix distances
ATOMS 34
C1P 6  0.05227  0.09614  0.35201 smb 193 1 0 5 4 3 2 0 0 0 0
C2P 6  0.50790 -0.42486  1.73105 smb 193 1 0 33 1 13 34 0 0 0 0
C7aP 6  1.14646 -0.18958 -0.67615 smb 193 1 3 10 8 1 0 0 0 0 0
C1a 6 -1.43540 -0.15284 -0.09901 smb 225 1 0 1 6 5 19 0 0 0 0
C6a 6 -0.90374  1.33585  0.19807 smb 225 1 0 1 12 4 20 0 0 0 0
C1b 6 -2.53245 -0.34516  0.95407 smb 65 1 3 9 7 4 0 0 0 0 0
C5a 6 -2.67471  0.78906  1.78434 smb 193 1 6 12 11 6 0 0 0 0 0
C3aP 6  2.18421 -0.89316 -0.02447 smb 193 1 5 14 13 3 0 0 0 0 0
C2 6 -3.33167 -1.49570  1.14964 smb 33 1 3 6 16 32 0 0 0 0 0
C7P 6  1.22297  0.13178 -2.05170 smb 33 1 3 3 17 31 0 0 0 0 0
C5 6 -3.62620  0.80578  2.83112 smb 33 1 3 7 15 30 0 0 0 0 0
C6 6 -1.69388  1.91193  1.39641 smb 97 1 0 21 5 7 22 0 0 0 0
C3P 6  1.87933 -1.12796  1.47367 smb 193 1 0 28 2 8 29 0 0 0 0
C4P 6  3.33544 -1.30143 -0.73873 smb 33 1 3 8 18 27 0 0 0 0 0
C4 6 -4.43617 -0.34370  3.03139 smb 33 1 3 11 16 26 0 0 0 0 0
C3 6 -4.28965 -1.48569  2.19774 smb 33 1 3 9 15 25 0 0 0 0 0
```

```
C6P 6  2.37685 -0.27708 -2.77101 smb 33 1 3 10 18 24 0 0 0 0 0
C5P 6  3.42305 -0.98792 -2.12156 smb 33 1 3 14 17 23 0 0 0 0 0
H1a_C1a 1 -1.66773 -0.28723 -1.15854 smb 22 1 0 4 0 0 0 0 0 0 0
H6a_C6a 1 -0.80132  1.93924 -0.70751 smb 22 1 0 5 0 0 0 0 0 0 0
Ha6_C6 1 -1.02622  2.15198  2.22783 smb 150 1 0 12 0 0 0 0 0 0 0
Hb6_C6 1 -2.24898  2.80522  1.09883 smb 150 1 0 12 0 0 0 0 0 0 0
H5P_C5P 1  4.30338 -1.29726 -2.69074 smb 22 1 0 18 0 0 0 0 0 0 0
H6P_C6P 1  2.46162 -0.04342 -3.83537 smb 22 1 0 17 0 0 0 0 0 0 0
H3_C3 1 -4.91758 -2.36425  2.36647 smb 22 1 0 16 0 0 0 0 0 0 0
H4_C4 1 -5.17659 -0.35041  3.83537 smb 22 1 0 15 0 0 0 0 0 0 0
H4P_C4P 1  4.13678 -1.84753 -0.23447 smb 22 1 0 14 0 0 0 0 0 0 0
Hb3P_C3P 1  2.67087 -0.69039  2.08738 smb 146 1 0 13 0 0 0 0 0 0 0
Ha3P_C3P 1  1.81579 -2.20114  1.67087 smb 146 1 0 13 0 0 0 0 0 0 0
H5_C5 1 -3.73121  1.68587  3.47071 smb 22 1 0 11 0 0 0 0 0 0 0
H7P_C7P 1  0.41703  0.67663 -2.54994 smb 22 1 0 10 0 0 0 0 0 0 0
H2_C2 1 -3.20959 -2.37248  0.50855 smb 22 1 0 9 0 0 0 0 0 0 0
Ha2P_C2P 1 -0.21396 -1.13404  2.14412 smb 146 1 0 2 0 0 0 0 0 0 0
Hb2P_C2P 1  0.63346  0.40798  2.42765 smb 146 1 0 2 0 0 0 0 0 0 0
SITE_NAMES
 2 C2P                    3 C7aP                   19 H1a_C1a                20
H6a_C6a                21 Ha6_C6
 22 Hb6_C6                23 H5P_C5P                24 H6P_C6P                25
H3_C3                  26 H4_C4
 27 H4P_C4P               28 Hb3P_C3P               29 Ha3P_C3P               30
H5_C5                  31 H7P_C7P
 32 H2_C2                 33 Ha2P_C2P               34 Hb2P_C2P
DD_TENSORS 12
19 4    1.00000    1.00000   -2.00000    0.00000    0.00000    0.00000(2)
20 5    0.10000    0.10000   -0.20000    0.00000    0.00000    0.00000(2)
21 12    0.95000    0.95000   -1.90000    0.00000    0.00000    0.00000(1)
22 12    1.75000    1.75000   -3.50000    0.00000    0.00000    0.00000(1)
23 18   -3.20000   -3.20000    6.40000    0.00000    0.00000    0.00000(1)
24 17    0.62500    0.62500   -1.25000    0.00000    0.00000    0.00000(1.5)
25 16    0.15000    0.15000   -0.30000    0.00000    0.00000    0.00000(1)
26 15   -2.45000   -2.45000    4.90000    0.00000    0.00000    0.00000(1)
```

```
27 14    0.85000    0.85000   -1.70000    0.00000    0.00000    0.00000(1)
30 11    1.02500    1.02500   -2.05000    0.00000    0.00000    0.00000(2)
31 10    0.55000    0.55000   -1.10000    0.00000    0.00000    0.00000(1)
32 9    1.10000    1.10000   -2.20000    0.00000    0.00000    0.00000(2)
FIXED_DISTANCES 8
2 4 2.68319 5 2.72816
3 4 2.64583 5 2.70084
21 5 2.19112 7 2.18443 22 1.78788
22 5 2.18634 7 2.17165
28 2 2.20815 8 2.17666
29 2 2.20666 8 2.17272 28 1.78522
33 1 2.18995 13 2.19805 34 1.78223
34 1 2.17791 13 2.19576
```

Data file for COSMOS MD simulation (.cod) for spiroindene (2)

The cosmos data file (*.cod*) containing the experimental data for spiroindene prepared for the MDOC run in the program COSMOS is shown below. The file contains at the beginning lines with remarks (REMARK), which contains information about the conditions and the origin of data, the used groups and their meaning. The experimental RDCs used as constraints follow (DD_TENSORS), showing the used grouping (GROUP_MEMBERS), and fixing of the methylene protons (FIXED_DISTANCES). In this case an extra fixing is required to maintain the hybridisation of the carbon at the spiro position.

Beginning of the COSMOS data file

```
$COD003
REMARK Dipolar Couplings for Spiroindene taken from J.Am.Chem.Soc. 126 (2004),
45, 14690-14691.
REMARK "Stretched Polydimethylsiloxane Gels as NMR-Alignment Media for Apolar and
Weakly Polar Organic Solvents: Ideal tool for Measuring RDCs at Low Molecular
Concentrations"
REMARK Errors taken from PALES Data File
REMARK DDs added
REMARK Group 1,2,3,5,6,7,8 added
REMARK added Spirocarbons C2P, C7aP, C[16]a for avoiding flipping into group 8
(former methylene fixing)
REMARK V1.1
RC_OPT_MOLECULE IN_MOL
RDC_OPT_MOLECULE IN_MOL
NAMES_OPT_FIT_TO EXACT
DATA_OPT_END
DD_TENSORS 12
H3_C3 C3       0.15   0.15   -0.30  0 0 0(1.0)
H4_C4 C4       -2.45  -2.45  4.90   0 0 0(1.0)
H5_C5 C5       1.025  1.025  -2.05  0 0 0(2.0)
H2_C2 C2       1.10   1.10   -2.20  0 0 0(2.0)
H1a_C1a C1a    1.00   1.00   -2.00  0 0 0(2.0)
Ha6_C6 C6      0.95   0.95   -1.90  0 0 0(1.0)
Hb6_C6 C6      1.75   1.75   -3.50  0 0 0(1.0)
H6a_C6a C6a    0.10   0.10   -0.20  0 0 0(2.0)
H4P_C4P C4P    0.85   0.85   -1.70  0 0 0(1.0)
H5P_C5P C5P    -3.20  -3.20  6.40   0 0 0(1.0)
H6P_C6P C6P    0.625  0.625  -1.25  0 0 0(1.5)
H7P_C7P C7P    0.55   0.55   -1.10  0 0 0(1.0)
FIXED_DISTANCES 9
H[ab]2P_C2P    C[13]P
Ha2P_C2P       Hb2P_C2P
H[ab]3P_C3P    C3aP
H[ab]3P_C3P    C2P
Ha3P_C3P       Hb3P_C3P
H[ab]6_C6      C[56]a
```

```
Ha6_C6         Hb6_C6
C7aP           C[16]a
C2P            C[16]a
GROUP_MEMBERS 22
C.*$                 1 all Carbons
H.*$                 2 all Protons
H[2-5]_C[2-5]        3 DDs
H[ab]6_C6            3
H[16]a_C[16]a        3
H[4-7]P_C[4-7]P      3
H.*$                 5 Fix CH-bond
C[16]a               6 13C for RDC calculation
C[2-6]               6
C[4-7]P              6
C1[abP]              7 Torsion angles
C[23]P               7
C[37]aP              7
C[56]a               7
C6                   7
H[ab][23]P_C[23]P    8 Fix distances
C[123]P              8
C3aP                 8
H[ab]6_C6            8
C[56]a               8
C7aP                 8
C[16]a               8
END
```

End of COSMOS data file

Staurosporine

Experimental data for staurosporine

Table S3. Experimental data for staurosporine in dPS/$CDCl_3$ as obtained from [3]

	δ_{1H}/[ppm]	δ_{13C}/[ppm]	$^1T_{CH}$/[Hz]	$^1D_{CH}$/[Hz]	Error [Hz]
C3‘-H3‘	3.87	84.0	120.5	-19.8	5.0
C4’-H4’	3.33	50.5	131.5	-3.7	1.0
C5’-H5’a	2.70	30.1	139.2	7.5	3.2
C6’-H6’	6.52	80.2	165.1	5.8	1.1
C2’-HMe	2.37	30.0	126.0	-3.2	1.0
C1-H1	7.23	107.0	130.8	-27.2	2.5
C2-H2	7.43	125.2	147.0	-11.1	7.6
C3-H3	7.32	119.9	162.3	1.7	4.2
C4-H4	9.40	126.7	137.9	-27.1	1.0
C8-H8	7.83	120.8	132.8	-25.3	1.0
C9-H9	7.28	120.1	148.0	-12.9	5.4
C10-H10	7.38	124.3	162.2	2.6	3.6
C11-H11	7.88	115.2	137.1	-25.4	1.1

Coordinates of staurosporine (3) in pdb file format

The coordinates of the staurosporine **3** in a pdb-file format generated as an output from the program COSMOS with the correct configuration and a starting chair conformation is given below. This configuration is marked as *Ref* throughout the manuscript.

```
HEADER    NO HEADER DEFINED AND DEFAULT DATE USED        01-JAN-00
AUTHOR    PDB FILE FORMAT GENERATED BY COSMOS
CRYST1    1.000    1.000     1.000   90.000   90.000    90.000 4
COMPND
REMARK    CREATED FILE CONTAINS ONLY BASIC MOLECULAR DATA
REMARK CELL  1.0000  1.0000  1.0000  90.000  90.000  90.000  0.0000 -0.7736
0.3485
REMARK SPGROUP 4
REMARK OPERATION CFF geometry optimization
REMARK ENERGY 1746.24
REMARK FORCE_FIELD_ENERGIES BondEn=20.0897 AngleEn=524.996 TorsionEn=968.372
PiTorsionEn=2.24663 CoulombEn=154.058 VdWEn=76.4751
HETATM    1  O3p NOP A   1       7.313   3.655   0.511  1.00  0.00           O
HETATM    2  O5  NOP A   1      11.712   5.937   8.474  1.00  0.00           O
HETATM    3  O1  NOP A   1       9.895   6.354   1.022  1.00  0.00           O
HETATM    4  N4  NOP A   1       9.577   2.924   2.528  1.00  0.00           N
HETATM    5  N13 NOP A   1      11.343   5.716   2.952  1.00  0.00           N
HETATM    6  N12 NOP A   1       8.086   5.887   2.705  1.00  0.00           N
HETATM    7  N6  NOP A   1       9.398   6.034   8.610  1.00  0.00           N
HETATM    8  C   CH3 A   1       7.691   6.701   0.348  1.00  0.00           C
HETATM    9  CNM NOP A   1      10.555   1.995   3.146  1.00  0.00           C
HETATM   10  COM NOP A   1       6.788   2.914   1.634  1.00  0.00           C
HETATM   11  C6p NOP A   1      11.080   5.646   1.483  1.00  0.00           C
HETATM   12  C5p NOP A   1      11.078   4.219   0.886  1.00  0.00           C
HETATM   13  C4p NOP A   1       9.760   3.438   1.132  1.00  0.00           C
HETATM   14  C3p NOP A   1       8.597   4.338   0.611  1.00  0.00           C
HETATM   15  C2p NOP A   1       8.561   5.773   1.241  1.00  0.00           C
HETATM   16  C13 NOP A   1      12.592   5.581   3.547  1.00  0.00           C
HETATM   17  C12 NOP A   1      10.392   5.805   3.957  1.00  0.00           C
HETATM   18  C12 NOP A   1       8.930   5.882   3.827  1.00  0.00           C
HETATM   19  C11 NOP A   1       6.770   5.903   3.207  1.00  0.00           C
HETATM   20  C11 NOP A   1       5.452   5.873   2.602  1.00  0.00           C
HETATM   21  C10 NOP A   1       4.273   5.913   3.395  1.00  0.00           C
HETATM   22  C9  NOP A   1       4.328   5.947   4.806  1.00  0.00           C
HETATM   23  C8  NOP A   1       5.576   5.956   5.461  1.00  0.00           C
HETATM   24  C7c NOP A   1       6.776   5.939   4.665  1.00  0.00           C
HETATM   25  C7b NOP A   1       8.166   5.935   5.072  1.00  0.00           C
HETATM   26  C7a NOP A   1       8.800   5.957   6.372  1.00  0.00           C
HETATM   27  C7  NOP A   1       8.196   6.022   7.754  1.00  0.00           C
HETATM   28  C5  NOP A   1      10.552   5.953   7.923  1.00  0.00           C
HETATM   29  C4c NOP A   1      10.235   5.893   6.497  1.00  0.00           C
HETATM   30  C4b NOP A   1      11.043   5.785   5.271  1.00  0.00           C
HETATM   31  CA4 NOP A   1      12.467   5.634   4.995  1.00  0.00           C
HETATM   32  C4  NOP A   1      13.639   5.534   5.826  1.00  0.00           C
HETATM   33  C3  NOP A   1      14.904   5.376   5.214  1.00  0.00           C
```

```
HETATM   34  C2  NOP A   1      15.034   5.314   3.806  1.00  0.00           C
HETATM   35  C1  NOP A   1      13.898   5.411   2.958  1.00  0.00           C
HETATM   36  H   N4  A   1       9.458   3.707   3.177  1.00  0.00           H
HETATM   37  HMe NOP A   1       6.667   6.342   0.199  1.00  0.00           H
HETATM   38  HMe NOP A   1       7.646   7.720   0.761  1.00  0.00           H
HETATM   39  HMe NOP A   1       8.119   6.786  -0.663  1.00  0.00           H
HETATM   40  H6p NOP A   1      11.922   6.183   1.019  1.00  0.00           H
HETATM   41  H5p NOP A   1      11.959   3.647   1.217  1.00  0.00           H
HETATM   42  H5p NOP A   1      11.197   4.327  -0.204  1.00  0.00           H
HETATM   43  H4p NOP A   1       9.794   2.540   0.493  1.00  0.00           H
HETATM   44  H3p NOP A   1       8.844   4.512  -0.449  1.00  0.00           H
HETATM   45  H11 NOP A   1       5.300   5.804   1.539  1.00  0.00           H
HETATM   46  H10 NOP A   1       3.308   5.911   2.908  1.00  0.00           H
HETATM   47  H9  NOP A   1       3.414   5.964   5.384  1.00  0.00           H
HETATM   48  H8  NOP A   1       5.614   5.972   6.540  1.00  0.00           H
HETATM   49  H7a NOP A   1       7.575   5.138   7.963  1.00  0.00           H
HETATM   50  H7b NOP A   1       7.597   6.935   7.896  1.00  0.00           H
HETATM   51  H6  NOP A   1       9.352   6.090   9.640  1.00  0.00           H
HETATM   52  H4  NOP A   1      13.570   5.575   6.902  1.00  0.00           H
HETATM   53  H3  NOP A   1      15.787   5.301   5.833  1.00  0.00           H
HETATM   54  H2  NOP A   1      16.014   5.190   3.368  1.00  0.00           H
HETATM   55  H1  NOP A   1      14.027   5.359   1.886  1.00  0.00           H
HETATM   56  HNM NOP A   1      10.687   1.093   2.530  1.00  0.00           H
HETATM   57  HNM NOP A   1      10.199   1.677   4.137  1.00  0.00           H
HETATM   58  HOM NOP A   1       7.357   1.988   1.803  1.00  0.00           H
HETATM   59  HOM NOP A   1       5.754   2.614   1.411  1.00  0.00           H
HETATM   60  HOM NOP A   1       6.773   3.481   2.572  1.00  0.00           H
HETATM   61  HNM NOP A   1      11.539   2.469   3.284  1.00  0.00           H
CONECT    1   10   14
CONECT    2   28
CONECT    3   11   15
CONECT    4   36    9   13
CONECT    5   11   16   17
CONECT    6   15   18   19
CONECT    7   51   27   28
CONECT    8   37   38   39   15
CONECT    9   61   56    4   57
CONECT   10   59   58   60    1
CONECT   11   40   12    5    3
CONECT   12   41   42   11   13
CONECT   13   43   12   14    4
CONECT   14   44   13   15    1
CONECT   15    8   14    6    3
CONECT   16   31   35    5
CONECT   17   18   30    5
CONECT   18   17   25    6
CONECT   19   20   24    6
CONECT   20   45   19   21
CONECT   21   46   20   22
CONECT   22   47   21   23
CONECT   23   48   22   24
CONECT   24   19   23   25
CONECT   25   18   24   26
CONECT   26   25   27   29
CONECT   27   49   50   26    7
```

```
CONECT   28   29    7    2
CONECT   29   26   28   30
CONECT   30   17   29   31
CONECT   31   16   30   32
CONECT   32   52   31   33
CONECT   33   53   32   34
CONECT   34   54   33   35
CONECT   35   55   16   34
CONECT   36    4
CONECT   37    8
CONECT   38    8
CONECT   39    8
CONECT   40   11
CONECT   41   12
CONECT   42   12
CONECT   43   13
CONECT   44   14
CONECT   45   20
CONECT   46   21
CONECT   47   22
CONECT   48   23
CONECT   49   27
CONECT   50   27
CONECT   51    7
CONECT   52   32
CONECT   53   33
CONECT   54   34
CONECT   55   35
CONECT   56    9
CONECT   57    9
CONECT   58   10
CONECT   59   10
CONECT   60   10
CONECT   61    9
MASTER        0    0    0    0    0    0    0    0
END
```

Coordinates of staurosporine in coo-file format (correct configuration)

The coordinates of staurosporine **3** in a coo-file format generated as an input for the program COSMOS with the correct configuration is given below.

```
$COO006
CELL  1.0000  1.0000  1.0000  90.000  90.000  90.000  0.0000 -0.7736  0.3485
NAME
SPGROUP 4
OPERATION CFF geometry optimization
ENERGY 1746.24
FORCE_FIELD_ENERGIES BondEn=20.0897 AngleEn=524.996 TorsionEn=968.372
PiTorsionEn=2.24663 CoulombEn=154.058 VdWEn=76.4751
ATOMS 61
HNMeb 1 11.53862  2.46940  3.28352 mb 1 0 40 0 0 0 0 0 0 0
HOMeb 1  6.77338  3.48126  2.57187 mb 1 0 38 0 0 0 0 0 0 0
HOMea 1  5.75423  2.61411  1.41077 mb 1 0 38 0 0 0 0 0 0 0
HOMec 1  7.35669  1.98770  1.80293 mb 1 0 38 0 0 0 0 0 0 0
O1 8  9.89456  6.35378  1.02193 mb 1 0 36 32 0 0 0 0 0 0
O5 8 11.71177  5.93743  8.47396 mb 1 1 19 0 0 0 0 0 0 0
O3p 8  7.31311  3.65458  0.51115 mb 1 0 38 33 0 0 0 0 0 0
N6 7  9.39828  6.03353  8.61005 mb 1 4 46 20 19 0 0 0 0 0
N12 7  8.08577  5.88667  2.70548 mb 1 6 32 29 28 0 0 0 0 0
N13 7 11.34322  5.71635  2.95222 mb 1 6 36 31 30 0 0 0 0 0
N4 7  9.57722  2.92385  2.52835 mb 1 0 61 40 34 0 0 0 0 0
C1 6 13.89846  5.41126  2.95766 mb 1 6 42 31 13 0 0 0 0 0
C2 6 15.03401  5.31352  3.80607 mb 1 6 43 14 12 0 0 0 0 0
C3 6 14.90378  5.37623  5.21389 mb 1 6 44 15 13 0 0 0 0 0
C4 6 13.63880  5.53381  5.82603 mb 1 6 45 16 14 0 0 0 0 0
Ca4 6 12.46744  5.63434  4.99486 mb 1 7 31 17 15 0 0 0 0 0
C4b 6 11.04300  5.78521  5.27124 mb 1 7 30 18 16 0 0 0 0 0
C4c 6 10.23506  5.89282  6.49702 mb 1 7 21 19 17 0 0 0 0 0
C5 6 10.55226  5.95316  7.92334 mb 1 7 18 8 6 0 0 0 0 0
```

```
C7 6  8.19571  6.02202  7.75381 mb 1 0 48 47 21 8 0 0 0 0
C7a 6  8.80035  5.95702  6.37238 mb 1 5 22 20 18 0 0 0 0 0
C7b 6  8.16582  5.93493  5.07192 mb 1 7 29 23 21 0 0 0 0 0
C7c 6  6.77588  5.93943  4.66485 mb 1 7 28 24 22 0 0 0 0 0
C8 6  5.57648  5.95609  5.46090 mb 1 6 49 25 23 0 0 0 0 0
C9 6  4.32782  5.94730  4.80575 mb 1 6 50 26 24 0 0 0 0 0
C10 6  4.27318  5.91276  3.39523 mb 1 6 51 27 25 0 0 0 0 0
C11 6  5.45218  5.87265  2.60168 mb 1 6 52 28 26 0 0 0 0 0
C11a 6  6.77022  5.90283  3.20691 mb 1 7 27 23 9 0 0 0 0 0
C12a 6  8.92992  5.88166  3.82671 mb 1 7 30 22 9 0 0 0 0 0
C12b 6 10.39241  5.80508  3.95652 mb 1 7 29 17 10 0 0 0 0 0
C13a 6 12.59214  5.58143  3.54744 mb 1 7 16 12 10 0 0 0 0 0
C2p 6  8.56110  5.77270  1.24056 mb 1 0 37 33 9 5 0 0 0 0
C3p 6  8.59718  4.33755  0.61086 mb 1 0 53 34 32 7 0 0 0 0
C4p 6  9.75972  3.43772  1.13198 mb 1 0 54 35 33 11 0 0 0 0
C5p 6 11.07816  4.21886  0.88642 mb 1 0 56 55 36 34 0 0 0 0
C6p 6 11.08000  5.64643  1.48341 mb 1 0 57 35 10 5 0 0 0 0
C_CH3 6  7.69127  6.70084  0.34794 mb 1 0 60 59 58 32 0 0 0 0
COMe 6  6.78839  2.91377  1.63405 mb 1 0 3 4 2 7 0 0 0 0
HNMec 1 10.19885  1.67718  4.13704 mb 1 0 40 0 0 0 0 0 0 0
CNMe 6 10.55529  1.99528  3.14603 mb 1 0 1 41 11 39 0 0 0 0
HNMea 1 10.68683  1.09309  2.53037 mb 1 0 40 0 0 0 0 0 0 0
H1 1 14.02724  5.35864  1.88623 mb 1 0 12 0 0 0 0 0 0 0
H2 1 16.01431  5.18968  3.36817 mb 1 0 13 0 0 0 0 0 0 0
H3 1 15.78691  5.30144  5.83253 mb 1 0 14 0 0 0 0 0 0 0
H4 1 13.56981  5.57471  6.90182 mb 1 0 15 0 0 0 0 0 0 0
H6 1  9.35233  6.08980  9.64007 mb 1 0 8 0 0 0 0 0 0 0
H7b 1  7.59713  6.93464  7.89570 mb 1 0 20 0 0 0 0 0 0 0
H7a 1  7.57474  5.13756  7.96316 mb 1 0 20 0 0 0 0 0 0 0
H8 1  5.61400  5.97175  6.53962 mb 1 0 24 0 0 0 0 0 0 0
H9 1  3.41441  5.96374  5.38356 mb 1 0 25 0 0 0 0 0 0 0
H10 1  3.30828  5.91062  2.90785 mb 1 0 26 0 0 0 0 0 0 0
H11 1  5.30049  5.80408  1.53902 mb 1 0 27 0 0 0 0 0 0 0
H3p 1  8.84394  4.51151 -0.44933 mb 1 0 33 0 0 0 0 0 0 0
```

```
H4p 1  9.79422  2.54025  0.49306 mb 1 0 34 0 0 0 0 0 0 0
H5pb 1 11.19706  4.32689 -0.20425 mb 1 0 35 0 0 0 0 0 0 0
H5pa 1 11.95853  3.64714  1.21727 mb 1 0 35 0 0 0 0 0 0 0
H6p 1 11.92192  6.18268  1.01942 mb 1 0 36 0 0 0 0 0 0 0
HMeb 1  8.11947  6.78564 -0.66322 mb 1 0 37 0 0 0 0 0 0 0
HMea 1  7.64650  7.72028  0.76100 mb 1 0 37 0 0 0 0 0 0 0
HMec 1  6.66739  6.34212  0.19925 mb 1 0 37 0 0 0 0 0 0 0
H_N4 1  9.45793  3.70706  3.17692 mb 1 0 11 0 9 0 0 0 0 0
```

Data file for MD simulation in program COSMOS for staurosporine 3

The cosmos data file (*.cod*) containing the experimental data for norcamphor prepared for the MDOC run in the program COSMOS is shown below. The file contains at the beginning lines with remarks (REMARK), which contains information about the conditions and the origin of data, the used groups and their meaning. The experimental RDCs used as constraints follow (DD_TENSORS), showing the used grouping (GROUP_MEMBERS), and fixing of the methylene protons (FIXED_DISTANCES).

<u>Beginning of the COSMOS data file</u>

```
$COD003
REMARK Staurosporine_.cod: modelling setup for the RDC constrained MD runs for 8
different Staurosporine starting coordinates:
REMARK SRRR, SRSR, SSRR, SSSR in chair and boat conformation
REMARK Dipolar 1 bond CH couplings and errors taken from
"ChemmCommun_08_5722_Luy_dPS_Staurosporin_SI.pdf"
REMARK "Deuterated polymer gels for measuring anisotropic NMR parameters with
strongly reduced artefacts" by Grit Kummerloewe
REMARK Fixing: - for fixing all initial CH-bond distances during run, in Force
Field Options, set "group 5" in "MD:Fix Bonds Lengths in Group"
REMARK  - for fixing (non-bond) distances in between geminal protons and to
second neighbours , set "group 8" in "MD:Fix Distances for Group"
REMARK  - for fixing all atoms to second neighbours, apply python script on coo-
File
REMARK Version: 1.0
RC_OPT_MOLECULE IN_MOL
RDC_OPT_MOLECULE IN_MOL
NAMES_OPT_FIT_TO EXACT
DATA_OPT_END
DD_TENSORS 13
H3p     C3p     9.90   9.90   19.8  0 0 0(5.0)
H4p     C4p     1.85   1.85   -3.7  0 0 0(1.0)
H5pa    C5p    -3.75  -3.75    7.5  0 0 0(3.2)
H6p     C6p    -2.90  -2.90    5.8  0 0 0(1.1)
C_CH3   C2p     1.60   1.60   -3.2  0 0 0(1.0)
H1      C1     13.60  13.60  -27.2  0 0 0(2.5)
H2      C2      5.55   5.55  -11.1  0 0 0(7.6)
H3      C3     -0.85  -0.85    1.7  0 0 0(4.2)
H4      C4     13.55  13.55  -27.1  0 0 0(1.0)
H8      C8     12.65  12.65  -25.3  0 0 0(1.0)
H9      C9      6.45   6.45  -12.9  0 0 0(5.4)
H10     C10    -1.30  -1.30    2.6  0 0 0(3.6)
H11     C11    12.70  12.70  -25.4  0 0 0(1.1)
FIXED_DISTANCES 4
H7[ab]  N6
```

```
H7[ab]  C7a
H7a     H7b
H5p[ab] C[46]p
GROUP_MEMBERS 23
C.*$                 1 all Carbons
H.*$                 2 all Protons
H[346]p              3 DDs
C_CH3                3
H5pa                 3
H[1-489]             3
H1[01]               3
H.*$                 5 Fix CH-bond
C[3-6]p              6 13C for RDC calculation3
[CH][1-489]          6
[CH][1][01]          6
O[13].*$             7 Torsion angles
C[2-6]p              7
N1[23]               7
N4                   7
C_CH3                7
C12[ab]              7
C[ON]Me              7
N6                   8 Fix distances of geminal protons
H7[ab]               8
C7a                  8
C[46]p               8
H5p[ab]              8
END
```

End of COSMOS data file

……

Oidiolactone B

Assignment data of Oidiolactone B

A total of 8.1 mg of solid Oidiolactone B was dissolved in 0.55 ml DMSO-d_6, corresponding to a 48 mM isotropic solution. The NMR spectra needed for the assignment of Oidiolactone B are recorded on Bruker Avance II 600 MHz spectrometer equipped with a 5 mm BBI inversely detected ^1H,^{13}C double resonance probehead with actively shielded z-gradient. The respective frequencies are 600.19 MHz for proton, and 150.92 MHz for carbon. The temperature is controlled with Bruker VT-unit and is set to 300K. The homonuclear experiments used for the structure elucidation are the following: 1D 1H experiment and standard 2D COSY, TOCSY and NOESY together with their 1D selective versions (sel. TOCSY and sel. NOE) with various mixing times. Heteronuclear experiments applied for assignment and RDC-measurement have been: modified 1D 13C experiment with a broadband BEBOP pulse for uniform excitation; a 2D CLIP-HSQC [4], a 2D P.E.HSQC [5], the HSQC-TOCSY-IP [6] and an HMBC. The aligned sample in PAN gel with 2.4 mm dry diameter of the polymer stick, irradiated with 440 kGy irradiation dose [7] consists of 12 mg analyte compound in 0.4 ml DMSO-d_6.

***Table S4**. Experimental data of the assignment for Oidiolactone B in DMSO-d_6*

Assignment	δ_{1H}/[ppm]	J_{HH}/[Hz]	δ_{13C}/[ppm]	Observed NOEs with
C1	-	-	162.42	
C2-H2	5.77 d	1.8	111.35	H5, w. H8, CH3-9, H10a, H10b
C3	-	-	157.38	
C4	-	-	132.48	
C5-H5	6.01 t	1.5	100.93	H2, H6, CH3-9, O-CH3
C6-H6	6.45 dt	4.6, 1.8	123.67	H5, H7, CH3-9, O-CH3
C7-H7	5.20 dt	4.8, 1.2	71.08	H6, H8, w. CH3-9, CH3-13
C8-H8	2.06 d	4.8	46.41	w. H2, H7, H12a, CH3-13
CH3-9	1.03 s	-	24.26	H2, H5, H6, H10b, H11*, H12b
C10-H10α	1.60 m*	q#, 7.3#	29.09	H2, H11*
C10-H10β	1.68 m*	t#, 6.7#	29.09	H2, CH3-9
C11-H11α	1.65 m*	m#, 7.1#	16.97	*

C11-H11β	1.65 m*	m#, 7.1#	16.97	*
C12-H12α	1.45 m	dd#, 7.0#, 6.7#	27.35	H8, CH3-13
C12-H12β	2.02 m	dd#, 7.3#, 5.4#	27.35	CH3-9, w. CH3-13
CH3-13	1.24 s	-	23.55	H7, H8, H11, H12a, w. H12b
C14	-	-	180.90	
O-CH3	3.59 s	-	56.70	H5, H6, H7

*- marks partially or fully overlapping signals. # indicates that multiplicity and the extracted coupling constants are derived from a P.E.HSQC experiment in isotropic solution.

Experimental data for Oidiolactone B in PAN/DMSO-d_6

***Table S5**. Experimental data for Oidiolactone B in PAN/DMSO-d_6 measured on a Bruker Avance II 600 MHz spectrometer at 300 K*

Assignment	δ_{1H}/[ppm]	δ_{13C}/[ppm]	$^{1}T_{CH}$/[Hz]	$^{1}D_{CH}$/[Hz]	Error [Hz]
C2-H2	5.77	111.35	160.45	-10.42	±0.4
C5-H5	6.01	100.93	177.68	5.77	±0.4
C6-H6	6.45	123.67	157.99	-10.13	±2.0
C7-H7	5.20	71.08	153.37	-0.76	±0.4
C8-H8	2.06	46.41	155.92	18.15	±1.0
CH3-9	1.03	24.26	123.35	-4.70	±1.0
C10-H10α	1.60	29.09	143.23	15.57	±1.5
C10-H10β	1.68	29.09	127.45	-1.26	±1.0
C11-H11α	1.65 *	16.97	128.84	0.52	±2.5
C11-H11β	1.65 *	16.97	130.83	0.76	±2.5
C12-H12α	1.45	27.35	128.41	-0.33	±1.0
C12-H12β	2.02	27.35	138.78	10.26	±1.5
CH3-13	1.24	23.55	129.78	0.42	±0.2
O-CH3	3.59	56.7	146.98	2.81	±0.4

*- marks partially or fully overlapping signals

Coordinates of Oidiolactone B in pdb file format

The coordinates of the Oidiolactone B **4** in a pdb-file format generated as an output from the program COSMOS with the correct configuration and a starting boat conformation is given below. This configuration is marked as *Ref* throughout the manuscript.

```
HEADER    NO HEADER DEFINED AND DEFAULT DATE USED        01-JAN-00
AUTHOR    PDB FILE FORMAT GENERATED BY COSMOS
COMPND     UNNAMED
REMARK    CREATED FILE CONTAINS ONLY BASIC MOLECULAR DATA
REMARK CELL  1.0000  1.0000  1.0000  90.000  90.000  90.000  0.0000  0.0000
0.0000
REMARK SPGROUP P1
REMARK SOURCE PDB: NO HEADER DEFINED AND DEFAULT DATE USED 01-JAN-00
CRYST1    1.000    1.000    1.000  90.00  90.00  90.00 P 1          1
ATOM      1  O14 A   A   1       2.149   4.162   2.097  1.00  0.00           O
ATOM      2  O1  A   A   1      -2.595   5.936  -0.905  1.00  0.00           O
ATOM      3  O14 NOP A   1       3.130   3.964   4.154  1.00  0.00           O
ATOM      4  O5  NOP A   1      -0.475   5.865  -1.911  1.00  0.00           O
ATOM      5  O1  NOP A   1      -4.610   6.160   0.088  1.00  0.00           O
ATOM      6  C13 CH3 A   1       1.065   1.613   3.792  1.00  0.00           C
ATOM      7  C5  CH3 A   1      -0.774   6.790  -2.947  1.00  0.00           C
ATOM      8  C9  CH3 A   1       0.046   6.167   3.302  1.00  0.00           C
ATOM      9  C10 NOP A   1      -1.771   4.635   4.122  1.00  0.00           C
ATOM     10  C11 NOP A   1      -1.176   3.741   5.224  1.00  0.00           C
ATOM     11  C12 NOP A   1       0.344   3.582   5.190  1.00  0.00           C
ATOM     12  C13 NOP A   1       0.858   3.137   3.811  1.00  0.00           C
ATOM     13  C8  NOP A   1      -0.042   3.598   2.663  1.00  0.00           C
ATOM     14  C9  NOP A   1      -0.815   4.926   2.933  1.00  0.00           C
ATOM     15  C3  NOP A   1      -1.514   5.302   1.614  1.00  0.00           C
ATOM     16  C4  NOP A   1      -0.671   5.288   0.419  1.00  0.00           C
ATOM     17  C6  NOP A   1       0.484   4.594   0.400  1.00  0.00           C
ATOM     18  C7  NOP A   1       0.937   3.699   1.508  1.00  0.00           C
ATOM     19  C14 NOP A   1       2.172   3.793   3.418  1.00  0.00           C
ATOM     20  C2  NOP A   1      -2.795   5.678   1.485  1.00  0.00           C
ATOM     21  C1  NOP A   1      -3.405   5.962   0.175  1.00  0.00           C
ATOM     22  C5  NOP A   1      -1.194   6.153  -0.709  1.00  0.00           C
ATOM     23  H10 C10 b   1      -2.113   5.578   4.571  1.00  0.00           H
ATOM     24  H10 C10 a   1      -2.681   4.132   3.769  1.00  0.00           H
ATOM     25  H11 C11 a   1      -1.627   2.743   5.138  1.00  0.00           H
ATOM     26  H11 C11 b   1      -1.484   4.121   6.206  1.00  0.00           H
ATOM     27  H12 C12 a   1       0.633   2.852   5.958  1.00  0.00           H
ATOM     28  H12 C12 b   1       0.818   4.512   5.518  1.00  0.00           H
ATOM     29  H3  C13 C   0       1.477   1.261   2.840  1.00  0.00           H
ATOM     30  H2  C13 C   0       0.121   1.085   3.965  1.00  0.00           H
ATOM     31  H1  C13 C   0       1.772   1.305   4.571  1.00  0.00           H
ATOM     32  H2  C2  A   1      -3.481   5.743   2.321  1.00  0.00           H
ATOM     33  H5  C5  A   1      -1.037   7.210  -0.449  1.00  0.00           H
ATOM     34  H3  C5  C   0      -0.171   6.530  -3.821  1.00  0.00           H
ATOM     35  H2  C5  C   0      -0.517   7.809  -2.641  1.00  0.00           H
ATOM     36  H1  C5  C   0      -1.830   6.735  -3.227  1.00  0.00           H
```

```
ATOM     37  H6  C6  A   1       1.124   4.628  -0.479  1.00  0.00           H
ATOM     38  H7  C7  A   1       1.135   2.710   1.076  1.00  0.00           H
ATOM     39  H8  C8  A   1      -0.789   2.824   2.428  1.00  0.00           H
ATOM     40  H2  C9  C   0      -0.587   7.061   3.384  1.00  0.00           H
ATOM     41  H1  C9  C   0       0.812   6.397   2.554  1.00  0.00           H
ATOM     42  H3  C9  C   0       0.558   6.076   4.260  1.00  0.00           H
CONECT    1   18   19
CONECT    2   21   22
CONECT    3   19
CONECT    4   22    7
CONECT    5   21
CONECT    6   29   30   31   12
CONECT    6
CONECT    7    4   34   35   36
CONECT    7
CONECT    8   40   41   42   14
CONECT    8
CONECT    9   23   24   10   14
CONECT    9
CONECT   10    9   25   26   11
CONECT   10
CONECT   11   10   27   28   12
CONECT   11
CONECT   12    6   11   13   19
CONECT   12
CONECT   13   12   39   14   18
CONECT   13
CONECT   14    8    9   13   15
CONECT   14
CONECT   15   14   16   20
CONECT   16   15   17   22
CONECT   17   16   37   18
CONECT   18    1   13   17   38
CONECT   18
CONECT   19    1    3   12
CONECT   20   15   32   21
CONECT   21    2    5   20
CONECT   22    2    4   16   33
CONECT   22
CONECT   23    9
CONECT   24    9
CONECT   25   10
CONECT   26   10
CONECT   27   11
CONECT   28   11
CONECT   29    6
CONECT   30    6
CONECT   31    6
CONECT   32   20
CONECT   33   22
CONECT   34    7
CONECT   35    7
CONECT   36    7
CONECT   37   17
CONECT   38   18
```

```
CONECT   39   13
CONECT   40    8
CONECT   41    8
CONECT   42    8
MASTER         0    0    0    0    0    0    0    0   42    0   42    0
END
```

Coordinates of Oidiolactone B in coo-file format

The coordinates of Oidiolactone B **4** in a coo-file format generated as an input for the program COSMOS with the correct configuration is given below.

```
$COO006
CELL  1.0000  1.0000  1.0000  90.000  90.000  90.000  0.0000  0.0000  0.0000
NAME  UNNAMED
SPGROUP P1
SOURCE PDB: NO HEADER DEFINED AND DEFAULT DATE USED 01-JAN-00
ATOMS 42
O1_a 8 -2.59528  5.93578 -0.90511 mb 1 0 10 9 0 0 0 0 0 0
O14_a 8  2.14968  4.16170  2.09650 mb 1 0 13 12 0 0 0 0 0 0
O1 8 -4.61038  6.16091  0.08793 mb 1 1 10 0 0 0 0 0 0 0
O5 8 -0.47512  5.86524 -1.91100 mb 1 0 9 7 0 0 0 0 0 0
O14 8  3.13045  3.96351  4.15361 mb 1 1 12 0 0 0 0 0 0 0
C9_CH3 6  0.04665  6.16757  3.30263 mb 1 0 42 41 40 17 0 0 0 0
C5_CH3 6 -0.77400  6.78989 -2.94726 mb 1 0 35 34 33 4 0 0 0 0
C13_CH3 6  1.06473  1.61261  3.79161 mb 1 0 31 30 29 19 0 0 0 0
C5 6 -1.19444  6.15327 -0.70878 mb 1 0 36 15 4 1 0 0 0 0
C1 6 -3.40492  5.96237  0.17470 mb 1 3 11 3 1 0 0 0 0 0
C2 6 -2.79500  5.67765  1.48435 mb 1 6 32 16 10 0 0 0 0 0
C14 6  2.17205  3.79258  3.41782 mb 1 2 19 5 2 0 0 0 0 0
C7 6  0.93653  3.69866  1.50788 mb 1 0 38 18 14 2 0 0 0 0
C6 6  0.48363  4.59406  0.40039 mb 1 2 37 15 13 0 0 0 0 0
C4 6 -0.67172  5.28885  0.41842 mb 1 3 16 14 9 0 0 0 0 0
C3 6 -1.51359  5.30207  1.61473 mb 1 6 17 15 11 0 0 0 0 0
C9 6 -0.81467  4.92563  2.93405 mb 1 0 22 18 16 6 0 0 0 0
C8 6 -0.04190  3.59785  2.66347 mb 1 0 39 19 17 13 0 0 0 0
C13 6  0.85835  3.13655  3.81122 mb 1 0 20 18 12 8 0 0 0 0
C12 6  0.34356  3.58107  5.19050 mb 1 0 28 27 21 19 0 0 0 0
C11 6 -1.17606  3.74056  5.22492 mb 1 0 26 25 22 20 0 0 0 0
```

```
C10 6 -1.77104  4.63467  4.12254 mb 1 0 24 23 21 17 0 0 0 0
H10_C10_a 1 -2.68089  4.13178  3.76918 mb 1 0 22 0 0 0 0 0 0 0
H10_C10_b 1 -2.11237  5.57796  4.57135 mb 1 0 22 0 0 0 0 0 0 0
H11_C11_b 1 -1.48414  4.12059  6.20576 mb 1 0 21 0 0 0 0 0 0 0
H11_C11_a 1 -1.62685  2.74285  5.13723 mb 1 0 21 0 0 0 0 0 0 0
H12_C12_b 1  0.81820  4.51160  5.51769 mb 1 0 20 0 0 0 0 0 0 0
H12_C12_a 1  0.63333  2.85163  5.95736 mb 1 0 20 0 0 0 0 0 0 0
H1_C13_CH3 1  1.77202  1.30416  4.57038 mb 1 0 8 0 0 0 0 0 0 0
H2_C13_CH3 1  0.12072  1.08499  3.96455 mb 1 0 8 0 0 0 0 0 0 0
H3_C13_CH3 1  1.47759  1.26068  2.84012 mb 1 0 8 0 0 0 0 0 0 0
H2_C2 1 -3.48162  5.74330  2.32019 mb 1 0 11 0 0 0 0 0 0 0
H1_C5_CH3 1 -1.82939  6.73490 -3.22785 mb 1 0 7 0 0 0 0 0 0 0
H2_C5_CH3 1 -0.51698  7.80866 -2.64129 mb 1 0 7 0 0 0 0 0 0 0
H3_C5_CH3 1 -0.17173  6.52957 -3.82162 mb 1 0 7 0 0 0 0 0 0 0
H5_C5 1 -1.03735  7.21007 -0.44872 mb 1 0 9 0 0 0 0 0 0 0
H6_C6 1  1.12364  4.62755 -0.47928 mb 1 0 14 0 0 0 0 0 0 0
H7_C7 1  1.13514  2.70995  1.07651 mb 1 0 13 0 0 0 0 0 0 0
H8_C8 1 -0.78939  2.82440  2.42821 mb 1 0 18 0 0 0 0 0 0 0
H3_C9_CH3 1  0.55792  6.07605  4.26072 mb 1 0 6 0 0 0 0 0 0 0
H1_C9_CH3 1  0.81200  6.39683  2.55427 mb 1 0 6 0 0 0 0 0 0 0
H2_C9_CH3 1 -0.58676  7.06036  3.38353 mb 1 0 6 0 0 0 0 0 0 0
```

Data file for COSMOS MD simulation (.cod) for oidiolactone B (4)

The cosmos data file (*.cod*) containing the experimental data for oidiolactone B **4** prepared for the MDOC run in the program COSMOS is shown below. The file contains at the beginning lines with remarks (REMARK), which contains information about the conditions and the origin of data, the used groups and their meaning. The experimental RDCs used as constraints follow (DD_TENSORS), showing the used grouping (GROUP_MEMBERS), and fixing of the methylene protons (FIXED_DISTANCES).

<u>Beginning of the COSMOS data file</u>

```
$COD003
REMARK NMR parameters for Oidiolactone B
REMARK Oidiolactone B in PAN and DMSO-d6
REMARK RDC from alignment with different stretching, here delta nu_Q=11 Hz,
clipHSQC data
REMARK Group 1: all Carbons
REMARK Group 2: all protons
REMARK Group 3: bond DD tensors - used as constraints (both atoms have to be in
group 1)
REMARK Group 4: RDC values - used as contraints (both atoms have to be in group
4)
REMARK Group 5: fixed distances
REMARK Group 6: 13C for RDC calculation
REMARK Group 7: Torsion angles for Mathematica snapshot
REMARK ordered exp. Values
RC_OPT_MOLECULE IN_MOL
RDC_OPT_MOLECULE IN_MOL
NAMES_OPT_FIT_TO EXACT
DATA_OPT_END
DD_TENSORS 14
H2_C2         C2    5.21         5.21   -10.42   0      0    0(0.4)
H5_C5         C5    -2.89        -2.89  5.77     0      0    0(0.4)
H6_C6         C6    5.07         5.07   -10.13    0     0    0(2.0)
H7_C7         C7    0.38         0.38   -0.76    0      0    0(0.4)
H8_C8         C8    -9.08        -9.08  18.15    0      0    0(1.0)
C9_CH3        C9    2.35         2.35   -4.70    0      0    0(1.0)
H10_C10_a     C10   -7.79        -7.79  15.57    0      0    0(1.5)
H10_C10_b     C10   0.63         0.63   -1.26    0      0    0(1.0)
H11_C11_a     C11   -0.26        -0.26  0.52     0      0    0(2.5)
H11_C11_b     C11   -0.38        -0.38   0.76    0      0    0(2.5)
H12_C12_a     C12   0.17         0.17   -0.33    0      0    0(1.0)
H12_C12_b     C12   -5.13        -5.13  10.26    0      0    0(1.5)
C13_CH3       C13   -0.21        -0.21  0.42     0      0    0(0.2)
C5_CH3        O5    -1.41        -1.41    2.81   0      0    0(0.4)
GROUP_MEMBERS 20
^C.*$               1 all Carbons
^H.*$               2 all Protons
H[2-8]_C[2-8]       3 DD tensor (constraints)
H1[0-2]_C1[0-2]_.*  3
```

```
C[59]_CH3             3
C13_CH3               3
H[2-8]_C[2-8]         4 RDC values
C[2-8]                4
H1[0-2]_C1[0-2]_.*    4
C1[0-3]               4
H[2-8].*_C[2-8]_.*    5 Atoms for fixed distances
^C[2-9]               6 13C for RDC calc
C1[0-3]               6
H[2567]_C[2-9]        7 tors angles
C[2-9]                7
H1[0-2]_C1[0-2]_.*    7
C1[0-3]               7
H1[0-2]_C1[0-2]_.*    8 Atoms for fixed distances
C1[0123]              8
C9                    8
FIXED_DISTANCES 6
H10_C10_.* C11
H10_C10_.* C9
H11_C11_.* C10
H11_C11_.* C12
H12_C12_.* C11
H12_C12_.* C13
END
```

End of COSMOS data file

References

1. *Merle, C., et al., Crosslinked poly(ethylene oxide) as a versatile alignment medium for the measurement of residual anisotropic NMR parameters. Angew Chem Int Ed Engl, 2013.* ***52****(39): p. 10309-12.*
2. *Freudenberger, J.C., et al., Stretched poly(dimethylsiloxane) gels as NMR alignment media for apolar and weakly polar organic solvents: an ideal tool for measuring RDCs at low molecular concentrations. J Am Chem Soc, 2004.* ***126****(45): p. 14690-1.*
3. *Kummerlowe, G., et al., Deuterated polymer gels for measuring anisotropic NMR parameters with strongly reduced artefacts. Chem Commun, 2008(44): p. 5722-4.*
4. *Enthart, A., et al., The CLIP/CLAP-HSQC: Pure absorptive spectra for the measurement of one-bond couplings. Journal of Magnetic Resonance, 2008.* ***192****(2): p. 314-322.*
5. *Tzvetkova, P., S. Simova, and B. Luy, PEHSQC: A simple experiment for simultaneous and sign-sensitive measurement of ((1)J(CH)+D-CH) and ((2)J(HH)+D-HH) couplings. Journal of Magnetic Resonance, 2007.* ***186****(2): p. 193-200.*
6. *Kobzar, K. and B. Luy, Analyses, extensions and comparison of three experimental schemes for measuring ((n)J(CH)+D-CH)-couplings at natural abundance. Journal of Magnetic Resonance, 2007.* ***186****(1): p. 131-141.*
7. *Kummerlowe, G., et al., Stretched poly(acrylonitrile) as a scalable alignment medium for DMSO. J Am Chem Soc, 2007.* ***129****(19): p. 6080-1.*
